# Remote Work, Well-Being, and Healthy Labor Force Participation Among Older Adults: A Scoping Review

**DOI:** 10.3390/ijerph22111719

**Published:** 2025-11-13

**Authors:** Kola Adegoke, Temitope Kayode, Mallika Singh, Michael Gusmano, Kenneth A. Knapp, Abigail M. Steger

**Affiliations:** 1 School of Health Sciences and Practice, New York Medical College, 40 Sunshine Cottage Road, Valhalla, NY 10595, USA; tkayode@student.touro.edu (T.K.); kenneth_knapp@nymc.edu (K.A.K.); asteger@student.touro.edu (A.M.S.); 2 College of Health, Lehigh University, Bethlehem, PA 18015, USA

**Keywords:** remote work, telework, older workers, aging workforce, labor force participation, healthy aging, flexible work arrangements, digital inclusion, occupational health, workforce retention

## Abstract

**Background:** Aging populations make expanded workforce participation among older adults an economic and public health priority. The COVID-19 pandemic accelerated the growth of virtual work, providing new opportunities for healthy aging in the workplace through increased flexibility and less physical strain. However, digital exclusion, ergonomically challenging tasks, and social isolation can limit these opportunities for older populations. **Objective:** This scoping review aimed to synthesize interdisciplinary research on the relationship between remote work and labor force participation among adults aged 45 years and older, focusing on health-related outcomes, barriers, and facilitators. **Methods:** Following the JBI Manual for Evidence Synthesis and PRISMA-ScR guidelines, we conducted a comprehensive search across seven databases for peer-reviewed and gray literature published between 2000 and 2025. Of 2108 records screened, 33 studies met the inclusion criteria. Data were extracted using a standardized charting tool and analyzed thematically. **Results:** Most studies were published after 2020 and originated in North America (45%) and Europe (40%). Core barriers included digital exclusion, ageism, and adverse ergonomic environments. Facilitators involved flexible working hours, a supportive organizational environment, and digital skills. Health-related outcomes such as stress reduction and improved well-being were commonly reported. However, only 18% of studies assessed policy effects, and very few examined intersectionality (e.g., gender, socioeconomic status). **Conclusions:** Remote and flexible work options can improve the health and participation of older adults in the workforce, but technology, infrastructure, and social barriers remain. Age-inclusive policies, digital equity efforts, and inclusive workplace practices are necessary to maximize the benefits of remote arrangements for aging populations.

## 1. Introduction

### 1.1. Background

The global population aged 60 and older is projected to double by 2050, reaching 2.1 billion [1,2]. This increasing demographic presents significant opportunities for the world’s public health system, labor market, and social policies. Encouraging active workforce participation among older adults has become a strategic goal in many countries, aiming to reduce pension burdens, address workforce shortages caused by aging, and prevent social exclusion later in life.

Meanwhile, flexible and remote work arrangements, driven by the COVID-19 pandemic, have changed how work is organized, especially in healthcare, education, and government administration. Older workers can benefit greatly from these setups, as they decrease physical stress, provide more independence, and help them manage illness or caregiving responsibilities. However, remote work can also worsen issues such as digital exclusion, reduced social interaction, and age-based discrimination in workplace technology and culture [3,4,5,6].

These complex dynamics are not well studied, especially among adults aged 45 and older. Although the World Health Organization defines older adults as those 60 or 65 years and above, we use a broader age range (45+) to include transitional midlife periods that are most relevant when examining detachment from the labor market, emerging chronic diseases, and retirement planning. This expanded age group allows for a more comprehensive analysis of the effects of remote work on populations entering or in older adulthood—key groups for preventive labor and health strategies.

Working remotely is also a new factor influencing health. Telework affects physical activity, ergonomic risks, stress, and social opportunities [7,8]. These aspects directly impact health outcomes and future functional abilities. In line with the Ottawa Charter for Health Promotion, creating supportive environments, improving health literacy, and implementing system-level policy changes are vital for advancing well-being throughout life [9]. When poorly executed, remote work can harm health and participation; when well-supported, it can enhance autonomy and resilience. Telework affects physical activity, ergonomic risks, stress, and social opportunities [7,8]. These aspects directly impact health outcomes and future functional abilities. In line with the Ottawa Charter for Health Promotion, creating supportive environments, improving health literacy, and implementing system-level policy changes are vital for advancing well-being throughout life [9]. When poorly executed, remote work can harm health and participation; when well-supported, it can enhance autonomy and resilience.

Furthermore, the United Nations Decade of Healthy Ageing (2021–2030) encourages the development of age-inclusive work practices that enhance both the quality and duration of life [10]. In this context, it is crucial to understand how remote work arrangements impact older adults’ engagement, health, and equity outcomes as we pursue the objectives of public health and labor policy.

**Figure 1:**
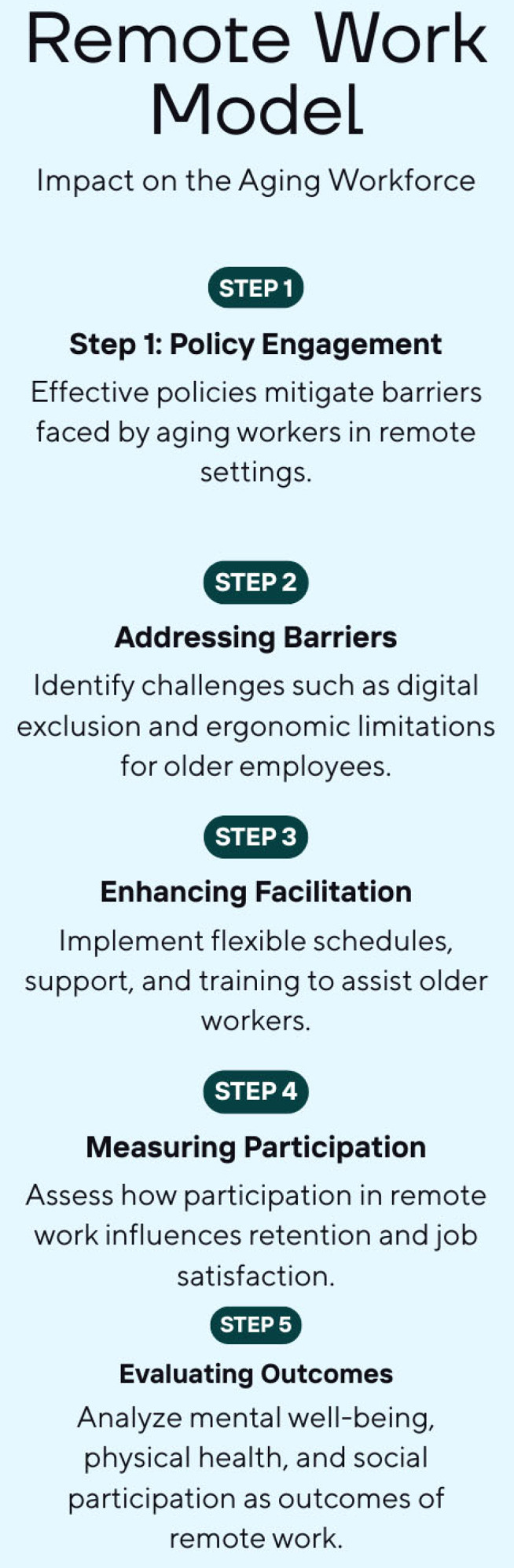
Conceptual framework illustrating the relationship between remote work and labor force participation among older adults.

To inform our synthesis, we created a conceptual framework (Figure 1) based on established models and existing literature in aging, digital equity, workforce flexibility, and workplace health [11,12,13]. The framework emphasizes barriers (e.g., digital exclusion, ergonomic limitations, ageism) and facilitators (e.g., flexible schedules, access to training, supportive managers) that affect older adults’ experiences with remote work. These elements influence both individual well-being and broader workforce retention trends. To promote healthy and sustainable aging in remote work settings, policy changes, workplace designs, and digital inclusion initiatives are essential tools.

The five-step model illustrates theoretical and empirical links discussed in early aging and occupational health literature [3,5,6]. Created using Canva 2.335.1 (Canva Pty Ltd.,110 Kippax Street, Surry Hills, NSW 2010, Australia), the model shows how policy engagement, digital barriers, facilitation, and outcome evaluation interact in remote work settings for adults aged 45 and older.

### 1.2. Rationale and Research Objectives

Although some research has been carried out on older adults’ experiences with telework, there is no comprehensive evidence that identifies all the barriers, facilitators, and health effects of working from home for this group. Varying definitions, sector differences, and cross-country variations further complicate the evidence base.

To address these gaps, the current scoping review aims to systematically map and synthesize the interdisciplinary literature on the relationship between remote work and labor force participation among adults aged 45 and older. Specifically, it seeks to:

- Identify the main barriers and supports that affect older adults’ participation in remote work and their related well-being outcomes.
- Consider the health promotion opportunities of working remotely in later life, particularly concerning autonomy, ergonomically safe work environments, and social inclusion.
- Develop workplace policies and public programs that promote diverse, inclusive, and age-inclusive virtual work environments.

Addressing the links between public health, labor economics, and gerontology, this review provides new insights for researchers, practitioners, and policymakers aiming to promote healthy aging through fair workforce participation.

## 2. Methods

This scoping review adhered to the methodological guidelines in the Joanna Briggs Institute (JBI) Manual for Evidence Synthesis [11]. The report was organized according to the Preferred Reporting Items for Systematic Reviews and Meta-Analyses extension for Scoping Reviews, (PRISMA-ScR) [12]. Additionally, we incorporated methodological improvements from Levac et al. [13].

The search was performed in close collaboration with librarians at New York Medical College, who scoped the project, developed and optimized database-specific search strategies, executed and documented searches, and supported reproducibility. The screening and data management process was supported by Covidence systematic review software (Veritas Health Innovation, Melbourne, Australia), enabling independent screening, conflict resolution, and data extraction. Completed PRISMA-ScR Checklist attached (File S4).

### 2.1. Eligibility Criteria

Eligibility criteria were developed using the Population–Concept–Context (PCC) framework, as recommended for scoping reviews [11].

- **Population:** Individuals aged 45 and older. The cutoff at 45 years was chosen to reflect midlife changes relevant to workforce engagement and aligns with life-course perspectives in the literature on labor and aging.
- **Concept:** Flexible, hybrid, or remote work options such as telework, virtual work, working from home, or telecommuting.
- **Context:** Throughout the world, across all occupations, sectors, and income levels.

**Inclusion Criteria**: Studies were eligible if they:

- Were published between January 2000 and May 2025.
- Included peer-reviewed or gray literature (e.g., government reports, dissertations).
- Employed any study design (qualitative, quantitative, or mixed methods).
- Reported among adults aged 45 and older on at least one of the following:
  o Labor force participation;
  o Employment or retirement outcomes;
  o Job satisfaction;
  o Physical or mental health;
  o Inclusion or exclusion from workplace practices.

**Exclusion Criteria**: Studies were excluded if they:

- Did not report age-specific findings.
- Did not specify remote or flexible work contexts.
- Focused exclusively on interventions unrelated to labor force participation or work settings.

### 2.2. Search Strategy

The databases searched were the seven largest academic databases:

- MEDLINE (Ovid).
- EMBASE.
- Scopus.
- CINAHL (EBSCOHost).
- AgeLine (EBSCOHost).
- PsycINFO (EBSCOHost).
- EconLit.

Combined searches of MeSH, Emtree, and keyword terms such as aging, older workers, working remotely, flexible schedules, and labor participation. Boolean operators (AND, OR), truncation (*), and proximity operators (e.g., NEAR, adj, PRE/W) were used together to enhance both precision and sensitivity. Full search strategies are available in Supplementary File S1.

The searches were limited to English-language publications and supplemented with citation chaining and targeted gray literature searches.

### 2.3. Search Results

A total of 2108 records were identified: 2098 through databases and 10 through citation searching. After removing 369 duplicates, 1739 unique records were screened based on titles and abstracts; 1646 were excluded, and 93 were assessed in full. Sixty studies were excluded for reasons such as wrong indication (*n* = 49), intervention (*n* = 8), setting (*n* = 1), population (*n* = 1), or research question misalignment (*n* = 1). The remaining 33 studies met all inclusion criteria and were included in the final synthesis.

**Figure 2:**
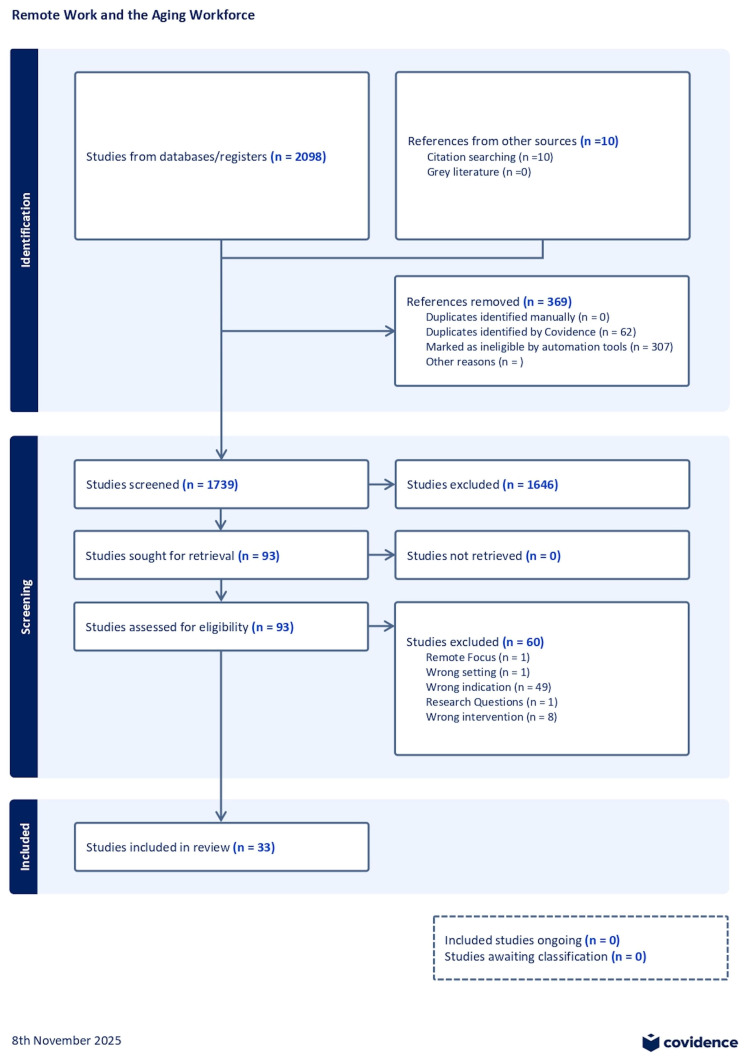
A PRISMA flow diagram presents the screening process and the number of studies included/excluded at each stage (Supplementary File S1).

The selection process is illustrated in Figure 2 (PRISMA 2020 flow diagram).

### 2.4. Study Selection Process

Extraction was carried out using [K.A.] and [T.K.], and manually checked by [M.S.] for consistency.

Study selection was performed using the Covidence software in a three-step screening process:

1. **Title and Abstract Screening:** Carried out by three reviewers individually.
2. **Full-Text Review:** Full-text articles that satisfied the initial inclusion criteria were reviewed against the full criteria.
3. **Discrepancy Resolution:** Differences were settled by discussion or by a third reviewer.

#### Reviewer Agreement and Discrepancies Resolution

- 116/1739 records (6.7%) needed reconciliation at the title/abstract level.
- 12/93 articles (12.9%) needed discussion at full-text review to reach consensus.

Inter-rater reliability was assessed for both the title/abstract and full-text screening. Agreement was high, with Cohen’s κ = 0.82 for title/abstract screening and κ = 0.79 for full-text screening, indicating substantial reliability between reviewers.

All disagreements were resolved through reviewer deliberation and, when an initial agreement was not reached, by consensus or referral to a third reviewer. These results indicate strong consensus, enhancing the reliability and transparency of the reviews.

### 2.5. Data Extraction and Charting

A standardized form for charting the data (see Supplementary Files S2 and S3) was created to abstract the following variables:

- Bibliographic details (author, year, country).
- Study design and method.
- Participant characteristics (age range, gender, job type).
- Remote work arrangement (telework, hybrid, flexible, etc.).
- Health or labor outcomes (e.g., well-being, inclusion, job satisfaction, retirement intentions).
- Identified barriers and facilitators.
- Policy or practice implications.

**Figure 3:**
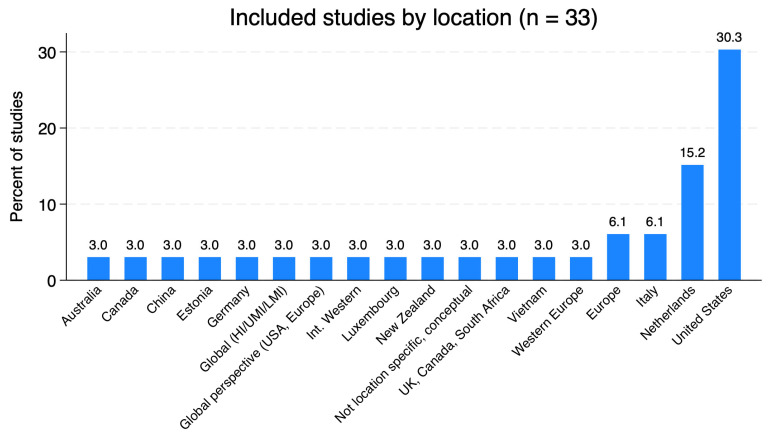
Geographic distribution of studies included in the review. Geographic distribution of included studies (*n* = 33) was generated using Stata 18 (StataCorp LLC, College Station, TX, USA). Bars show the percentage of included studies conducted in each country or region. “Global (HI/UMI/LMI)” denotes a multi-country study across high-, upper-middle- and lower-middle-income settings; “International Western” and “Western Europe” denote multi-country studies focused on Western, high-income contexts; “UK, Canada, South Africa” represents a single multi-country study; “Not location specific, conceptual” refers to a conceptual paper without a defined empirical setting. See Table 2 for underlying counts and percentages by country/region.

Data extraction was performed independently by several reviewers to establish reliability (Table 1 and Table 2). Descriptive statistics (e.g., frequencies and proportions of theme extraction) were generated in Stata 18 (StataCorp LLC, College Station, TX, USA) to assess thematic patterns across the included studies. These findings are tabulated (Figure 3).

### 2.6. Data Synthesis

The synthesis used a descriptive and thematic approach:

- **Quantitative summary:** The studies were summarized according to year, country, sector, design, and type of remote work.
- **Thematic analysis:** Key themes were derived related to:
- Barriers (e.g., digital exclusion, ageism, ergonomic risks);
- Facilitators (e.g., autonomy, training support, flexibility);
- Health promotion outcomes (e.g., mental health, well-being, and social participation).

Themes were organized in alignment with health promotion frameworks such as the Ottawa Charter for Health Promotion [9], which guided the interpretation of findings in terms of enabling environments, personal skills development, and supportive policies.

## 3. Results

### 3.1. Study Selection

A total of 2108 records were identified through database searches and citation chaining. Of these, 369 duplicates were removed. Then, 1739 records were screened based on title and abstract, and 93 were reviewed in full. Ultimately, 33 studies met the inclusion criteria (Figure 2).

### 3.2. Characteristics of Included Studies

The 33 studies, conducted between 2002 and 2025, covered diverse regions and research methods, focusing on remote or flexible work among adults aged 45 and older. Most took place in high-income countries such as the United States, Canada, and European nations, while one study in Vietnam provided an LMIC perspective. Methodologically, 15 studies were quantitative, 11 qualitative, 3 used mixed methods, 3 were conceptual reviews, and 1 was a systematic review. The populations studied varied, including contract workers [14], healthcare workers, teachers, and government employees, with focuses ranging from job performance to health and retirement transitions. European studies highlighted policy frameworks and digital infrastructure, whereas U.S. studies more often emphasized individual liberty and caregiving conflicts. A summary of the study designs and settings is available in Table 2 and Supplementary File S3.

Table 3 summarizes the characteristics, contexts, and findings of the 33 reviewed studies. An abridged version is shown for illustration; the whole table is available in Supplementary File S4 via OSF at https://doi.org/10.17605/OSF.IO/B6H7C. https://osf.io/tdgsv (accessed on 23 September 2025).

This methodological diversity reflects a balanced representation of empirical, theoretical, and policy-oriented perspectives on remote work and aging.

The population samples primarily comprised individuals aged 45 years and above, with subgroups including caregivers, teaching and healthcare professionals, government servants, and those based in specific geographic areas (Figure 3). The remote work arrangements explored included fully remote, hybrid, and flexible options.

Figure 3: This pie chart displays the geographic distribution of the 33 included studies. The United States accounts for the largest share, followed by Europe, North America, and a mix of global or unspecified locations.

### 3.3. Theme Coverage Across Studies

The coder coded studies for four major thematic areas: facilitators, barriers, policy implications, and health and labor outcomes. Text-based records were coded, and any incidence of descriptive data in the theme column was treated as a mention. The blank rows were assumed to indicate a lack of thematic discourse. Table 3 presents a quantitative summary.

Table 4: This table shows the percentage of studies (out of 33) that addressed each central theme. Barriers and facilitators were the most reported, followed closely by outcomes and policy implications.

#### Interpretation

Barriers and facilitators were the most consistently prominent themes, appearing in more than 90% of studies. This suggests a strong orientation toward identifying the structure and individual-level challenges and enablers in remote work for older adults. While the results and policymaking implications were also quite common (approximately 85% of the studies), a small proportion of studies were conceptual or theoretical and did not yield actionable results or policy implications.

### 3.4. Barriers

Barriers to remote work were multidisciplinary, covering digital, organizational, and psychosocial areas. Precarious contract workers experienced greater insecurity and fewer benefits than regular employees [14]. Psychological distress from sudden telework changes during the COVID-19 lockdown was common among older adults [15]. Inadequate ergonomics and technical issues limited satisfaction and well-being [16], while unclear expectations and increased workload reduced productivity [17].

Inflexible HR practices and a lack of personalized accommodations restricted older workers’ flexibility [18]. Organized workplace support was not always transformed into well-being, especially when relational bonds were weak [19]. Cognitive decline, digital gaps, and systemic discrimination were identified as overlooked aging factors in telework in broader reviews [20]. Gender disparities in workplace flexibility persisted, putting older women at a disadvantage [21]. Digital and social exclusion created compounded risks for individuals with limited networks or digital exposure [22].

Low digital confidence and technology anxiety limited participation, highlighting the need for formal digital training [23]. Blended or hybrid models were not always beneficial—older adults struggled with transitioning from in-person to remote roles [24]. In Germany, health-related employment restrictions reduced participation in flexible work arrangements [25]. Cross-age comparisons revealed that satisfaction with remote work heavily depended on trust in the employer and clear role definitions [26,27]. Systematic reviews found that poor workplace control and social isolation negatively affected mental and physical health [28,29]: limited digital skills and inconsistent managerial communication contributed to long-term inequalities [30,31].

Below is a cross-study comparison (Table 5) that summarizes the significant barriers and facilitators reported across the 33 included studies. Each category was derived by grouping similar themes from the extraction data; the “Number of studies” column indicates how many studies mentioned each category (out of 33).

Table 4: The most frequently reported barrier was digital exclusion, reflecting inadequate access to technology and skills among older adults. Ageism and employer bias were also common, indicating persistent stereotypes about older workers’ adaptability and a lack of supportive HR practices. Health limitations, cognitive strain, and structural and policy constraints further restricted older workers’ ability to engage in remote work.

### 3.5. Facilitators

Facilitators of autonomy, trust, and organizational culture include flexible schedules and phased retirements, which enabled older adults to continue participating [32]. Early research showed that telework could extend employability when supported by psychological and ergonomic measures [33]. Later studies found that formal digital onboarding increased satisfaction and inclusion among new and returning employees [34].

Federal agency telework programs showed increased retention when combined with flexible management and ergonomically designed tools [35]. The Vietnam case revealed that telework eased the decline in employment for some, while for others it worsened inequality based on connectivity levels and education [36]. Age-based boundary management tactics, such as separating work and home, were linked to productivity gains and better work–life balance [37].

Employer discretion in responding to aging workforces, such as through mentoring and flexible pensions plans, boosts retention and reduces turnover [38]. Infrastructure gaps in digital technology, especially in the public sector, restrict fair and equal opportunities for remote work [39]. Research linking flexibility to health shows improved well-being when organizational environments are supportive [40]. Smart use of intelligent technology decreased fatigue and increased satisfaction among older teleworkers [41].

Technology-supported ergonomic settings, such as ambient-assisted working systems, and augmented functional independence [42]. Positive attitudes toward technology and age-related cognitive adaptability help overcome age-based barriers to remote work [43]. Flexible work arrangements and organizational climates that foster trust improve health and productivity outcomes [44]. Virtual workplaces that emphasize self-efficacy and mentoring support “successful aging at work” [45]. Finally, integrating international HR standards and ethical considerations in technology enhances the long-term sustainability of an aging workforce [46].

Below is a cross-study comparison (Table 4) summarizing the significant barriers and facilitators reported across the 33 included studies. Each category was created by grouping similar themes from the extracted data; the “Number of studies” column shows how many studies mentioned each category (out of 33).

Table 6: The most common facilitators were flexible scheduling and a strong sense of community at work, both of which help older workers balance personal needs with professional demands. Digital upskilling programs and personalized job arrangements (Ideals) assist older adults in overcoming technology barriers and staying engaged [18]. Supportive organizational and policy environments, including phased retirement options, ergonomic adjustments, and inclusive leadership, also foster healthy and sustained participation in remote work.

### 3.6. Health and Labor Force Participation Outcomes

Health effects varied depending on the environment. Flexible and hybrid setups reduced commuting stress and enhanced mental health [14,16,19,25]. Conversely, technostress, social isolation, and boundary blurring increased cognitive fatigue and loneliness [15,22,27,29]. Workplace health benefits were most significant when both ergonomics and psychosocial supports were in place [40,41].

Labor participation trends showed delayed retirement and increased staying in caregiving and knowledge jobs [24,31,32]. Meanwhile, informal workers and women faced layered exclusion due to caregiving responsibilities and digital gaps [21,36]. Research reaffirmed the link between supportive policies, mentoring, and digital upskilling to support longer, healthier working lives [18,23,34,45].

### 3.7. Policy and Practice Implications

The evidence from the 33 studies shows that the success of remote work among older adults depends on equitable design. Organizations should provide personalized flexibility (“i-deals”) [18,32], digital training [23,30], ergonomic support [16,25], and age-inclusive mentoring [34,37]. Policymakers must expand digital infrastructure, protect informal workers, and redesign pension systems to support hybrid models [36,38,44]. Including international HR standards and responsible AI practices offers a way to sustain aging workforces in the long term [42,46].

**Figure 4:**
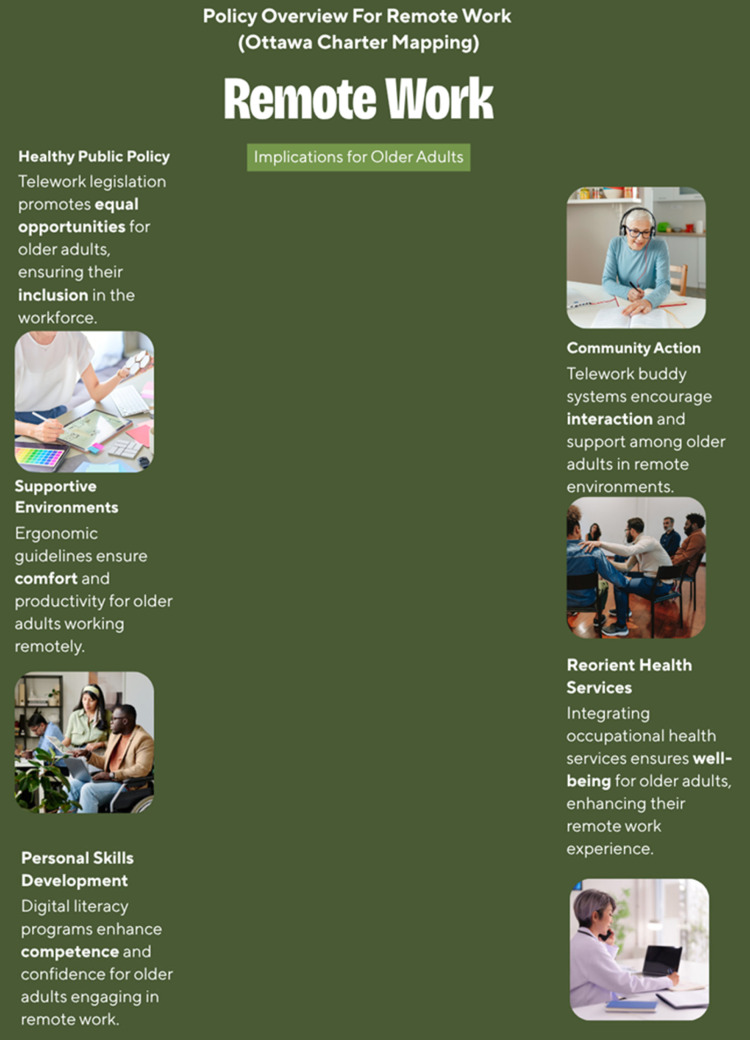
Policy Implications for Remote Work (Ottawa Charter Mapping), Infographic created using Canva (Canva Pty Ltd., 110 Kippax St, Surry Hills, NSW 2010, Australia).

These strategies encompassed the five areas of action outlined in the Ottawa Charter (Figure 4).

This diagram places “Remote Work for Older Adults” at the center and illustrates how recommended interventions align with the Charter’s action areas: building healthy public policy (telework legislation, age-sensitive incentives, digital equity); creating supportive environments (ergonomic guidelines, inclusive leadership, work–life boundaries); strengthening community action (telework buddy systems, peer and mentoring support, cross-generational learning); developing personal skills (digital literacy/upskilling, health programs, chronic-disease management); and reorienting health services (integration of occupational and mental health, telehealth and screenings, social insurance and counseling). The improved layout and consistent colors aim to present these relationships more clearly and professionally.

## 4. Discussion

### 4.1. Summary of Evidence

This review concludes that home-based work offers both structural potential and psychosocial risks for older workers. While flexible arrangements can support health and workforce retention, the distribution of benefits is uneven. Without strong policies and digital infrastructure, home-based work may lead to exclusion that worsens with age, especially among older workers with caregiving duties, chronic illnesses, or limited digital access [14,15,16,17,47]. Results should be viewed in light of the changing nature of home-based work. Studies from the early 2000s saw telework as the exception [18,20], whereas research after 2020 reflects more normalized arrangements, often imposed by regimes [48,49].

Telecommuting encourages continuous participation by reducing physical stress and helping older workers stay productive [14,17,47]. Despite these benefits, digital divides, limited institutional support, and ongoing ageism can weaken these advantages [16,22,48]. Organizational preparedness and inclusive HR practices, such as personalized flexibility agreements (i-deals) [18], technical assistance, ergonomic solutions [23], and age-inclusive management, are crucial to ensure remote work is empowering rather than exclusionary [19,21,45]. Without these support mechanisms, telecommuting may reinforce exclusion through information gaps, fewer mentorship opportunities, and technology-related disparities [22,48,50]. When executed well, remote work can enhance workforce participation, reduce healthcare costs, and increase economic productivity [17,24,49].

Older workers have stability, experience, and mentoring in increasingly fluid labor markets. They benefit GDP growth, reduce the pension burden, and promote intergenerational equity in the labor force.

### 4.2. Health Promotion Implications

The review highlights a gendered pattern. Working remotely can support older workers’ caregiving expectations, especially among older women, as working from home often overlaps with family caregiving, increasing unpaid labor and reinforcing traditional gender roles [47,51,52]. The combined influence of age, gender, and caregiving expectations underscores an urgent need for future research in occupational health. Remote work’s potential to reduce stress and enhance autonomy aligns with the Ottawa Charter’s emphasis on creating a supportive environment for stress reduction [9]. Flexible work options decrease commuting-related stress and improve work–life balance [19,24,27]. However, autonomy in remote work depends on proper ergonomics, digital literacy, and flexible management. Without these, telework can cause psychosocial stress instead of fostering empowerment [29,30,41]. Low-quality home environments and digital eye strain contribute to cognitive overload, while reduced social contact increases loneliness risk [22,29,52]. Therefore, the health benefits of telework depend on an individual’s capabilities and the adequacy of workplace infrastructure.

When properly supported, telework can extend working life, improve mental health, and enhance inclusion [14,19,27]. These benefits align with the WHO Decade of Healthy Ageing framework, which promotes environments that support older adults [48]. However, in settings where digital literacy is low or management is unsupportive, working from home or the office can widen inequality, particularly for people with long-term conditions [47,53].

### 4.3. Policy and Practice Relevance

Policies must consider the diversity of older workers. Age-friendly home-based work is not just about participation but also about removing structural barriers that prevent it—such as digital exclusion, inflexible pension systems, and deeply rooted workplace age discrimination [14,18,45,53]. A life-course approach is crucial, since digitalization affects different cohorts in different ways [50,51].

Organizational-level inclusive HR practices such as ICT-based onboarding, mentoring, and leader–employee participation have proven useful [18,23,34,40]. At the systemic level, restructuring labor and disability evaluation systems remains essential. For example, Italy’s OECD review highlighted the fragmentation of its disability evaluation system, which left many qualified older workers uncovered; using function-based tools such as the WHO Disability Assessment Schedule (WHODAS) can help improve inclusion [53].

Digitalization presents both opportunities and challenges. It enables flexible work arrangements and longer careers but demands lifelong learning and adaptability to technology [50,51]. Technology can empower as well as exclude, unless deliberate interventions are implemented [22,28,51]. Key enabling factors include flexible schedules, gradual retirements [19,24], digital upskilling [31], inclusive workplace cultures [18,23,34], and autonomy in team structures [53].

From this synthesis, several policy recommendations emerge:

- Digital inclusion: Governments and employers should provide targeted digital literacy programs, ergonomically designed tools, and reliable internet access [48,50].
- Anti-ageism measures: Recruitment, advancement, and preservation procedures should address stereotypes and include inclusive teachings [16,18].
- Pension modernization: Pension plans should support hybrid and part-time telework without reducing benefits for older workers [49].
- Health-adaptive job design: Work environments can incorporate ergonomic and psychosocial adaptations for older workers [47,54].

Successful policies balance personalized adaptability with organizational responsibility, ensuring that digital literacy, ergonomics support, and fairness are fundamental, not optional. 

These recommendations align with ISO 25550:2022, the international standard for age-inclusive workforce policies, which assesses flexible workforce arrangements, technology access, and age-sensitive HR practices as vital for older workers [55]. 

### 4.4. Research Gaps

Despite years of research, age-specific evidence remains limited outside high-income regions [47,48,53]. No studies including data from the Middle East or Sub-Saharan Africa were identified. Future research should focus on low- and middle-income settings where the informal economy and weaker digital infrastructure create unique challenges [51]. Variables such as gender, education, disability, and caregiving were reported inconsistently [47,50,51,52]. Older women, in particular, are often underrepresented despite facing dual caregiving and workforce duties [52]. This underrepresentation limits the ability of existing studies to inform equitable policies. Methodologically, most studies are cross-sectional and rely on self-reported proxies, such as satisfaction, rather than on health indicators [14,22,27]. Prospective and mixed-method research could help establish causal relationships among remote work, health, and labor participation. Expanding coverage to underrepresented regions and occupations would make telework policies more applicable globally [48,49,50,51]. 

### 4.5. Strengths and Limitations

The broad time span (2000–2025) allowed this review to incorporate evolving perspectives on remote work, from early pilot studies to post-pandemic normalization [18,48]. Its transdisciplinary approach, based on JBI and PRISMA-ScR templates [11,12,13], improves reliability. However, as a scoping review, no formal quality assessment was conducted, and variability in study design and outcomes makes direct comparisons difficult [14,15,16,17,18,19,20,21,22,23,24,25,26,27,28,29,30,31,32,33,34,35,36,37,38,39,40,41,42,43,44,45,46,47,48,53]. Many studies focused on intermediate rather than clinical health outcomes, which limits causal inference. Additionally, technological and policy changes over 25 years introduce contextual variability. Despite these challenges, consistent patterns emerged across disciplines, highlighting digital inclusion, workplace flexibility, and intergenerational equity as key elements of healthy aging in the future of work.

## 5. Conclusions

This scoping review demonstrates an urgent imperative to redefine flexible and remote work as a long-term solution for advancing healthy, inclusive, and productive aging at the workplace. Based on 33 studies mainly in high-income and international settings, the current evidence indicates that flexible working can contribute to greater autonomy, reduced physical stress, and improved work–life integration, particularly among older workers with chronic illness or caregiving obligations.

However, these benefits are not guaranteed. Without intentional investment in digital infrastructure, age-friendly technologies, and inclusive workplace policies, remote work can reinforce exclusion, widen digital divides, and increase inequalities, especially among workers with limited digital skills, disabilities, or informal employment.

Telework is not a neutral practice; its outcomes depend on the quality of technological access, leadership culture, and policy design. Persistent barriers, such as ageist attitudes, rigid pension systems, and inadequate training, continue to block equal participation. Employers and policymakers must respond proactively by expanding digital literacy programs, subsidizing connectivity costs and equipment, and incorporating anti-ageism measures into organizational and legal frameworks. Reforms in pensions, employment, and social care also need to support flexible, late-life work.

Economically, it helps older adults keep rural or hybrid jobs by increasing workforce resilience, reducing healthcare costs, and leveraging extensive experience, supported by both the WHO Decade of Healthy Ageing and the Ottawa Charter for Health Promotion. However, significant research gaps still exist, especially in low- and middle-income countries, and regarding intersectional issues such as gender, disability, and rurality.

Ultimately, telework can promote healthy and fair aging only when guided by intentional, evidence-based, and inclusive strategies. For employers, policymakers, and researchers, this presents both a responsibility and a chance to reshape the future of work, rooted in dignity, health, and intergenerational fairness.

## Figures and Tables

**Table 1:** Summary of extraction.

Stage	Count
Total Identified	2108
Removed (Duplicates)	369
Screened (Titles/Abstracts)	1739
Disagreements (Title/Abstract)	116
Excluded (Title/Abstract)	1646
Full Texts Reviewed	93
Disagreements (Full Text)	12
Excluded (Full Text)	60
Included in Review	33

**Table 2:** Geographic distribution of included studies (*n* = 33).

Country/region	*n*	%
United States	10	30.30
Netherlands	5	15.15
Europe (unspecified country)	2	6.06
Italy	2	6.06
Australia	1	3.03
Canada	1	3.03
China	1	3.03
Estonia	1	3.03
Germany	1	3.03
Luxembourg	1	3.03
New Zealand	1	3.03
Vietnam	1	3.03
Western Europe (multi-country)	1	3.03
UK, Canada, South Africa (multi-country)	1	3.03
Global perspective (high-, upper-middle-, and lower-middle-income)	1	3.03
Global perspective (USA, Europe)	1	3.03
International Western (multi-country)	1	3.03
Not location specific, conceptual	1	3.03
**Total**	**33**	**100.0**

*Note:* Percentages are based on the total number of included studies (*n* = 33) and may not sum exactly to 100% due to rounding. “Global (high-, upper-middle-, and lower-middle-income)” refers to a multi-country study spanning several World Bank income groups. “International Western” and “Western Europe” denote multi-country studies focused on Western, high-income settings. “Not location specific, conceptual” refers to a conceptual paper without a defined empirical location. “UK, Canada, South Africa” represents a single multi-country study.

**Table 3:** Summary of Reviewed Studies on Remote Work and Older Adults.

No	Citation	Year	Country	Income Level	Study Design	Population/Context	Key Barriers	Key Facilitators	Health/Labor Outcomes	Policy Implications
1	Abraham KG et al. J Pension Econ Fin 2021 [14]	2021	USA	High	Quantitative	Aged 50–79 contract workers	Ageism; few phased-retirement options	Contract flexibility	Bridge to retirement; income supplement	Promote flexible contract paths
2	Andreassi S et al. Healthcare 2021 [15]	2021	Italy	High	Mixed-methods	Older virtual workers	Emotional strain; isolation	Spiritual growth; engagement	Improved coping but higher stress variance	Targeted mental-health support
3	Arvola R et al. Sci Ann Econ Bus 2017 [16]	2017	Estonia	High	Survey	Aged 50+ teleworkers	ICT skill gaps	Flexible hours	Higher satisfaction; reduced commuting fatigue	Invest in digital training
4	Beekman EM et al. Work 2025 [17]	2025	The Netherlands	High	Cross-sectional	Gov’t teleworkers	Work pressure; role ambiguity	Clear communication	Sustained employability; reduced burnout	Define telework roles clearly
5	Bal PM & Jansen PGW [18]	2014	Europe	High	Conceptual	Older employees	Uniform HR rules	I-deals	Motivation and retention ↑	Individualize HR policies
6	Buonomo I et al. IJERPH 2023 [19]	2023	Italy	High	Cross-sectional	Remote workers	High demands; weak support	Sense of community	Job satisfaction ↑; stress moderated	Foster social connectedness
7	Czaja SJ & Sharit J [20]	2009	USA	High	Policy review	Aging workers	Age bias; obsolete training	Tech design; lifelong learning	Retention ↑; cognitive benefits	Modernize retirement systems
8	Damman M & Henkens K J Appl Gerontol 2020 [21]	2020	The Netherlands	High	Survey	Older employees 60–65	Gender gap in flexibility	Schedule autonomy	Work satisfaction ↑ for women	Tailor policies by gender
9	Seifert A et al. J Gerontol B 2021 [22]	2021	Switzerland	High	Quantitative	Older adults	Digital/social exclusion	ICT access	Improved social connectedness	Close digital divide
10	Dijkstra K Gerontechnology 2024 [23]	2024	The Netherlands	High	RCT	Aged 50+ workers	Cognitive decline	Digital training	Better memory & attention scores	Invest in cognitive upskilling
11	Dropkin J et al. Work, Aging and Retirement 2016 [24]	2016	USA	High	Conceptual	Older teleworkers	Policy gaps	Ergonomic support	Extended employment tenure	Adopt blended work models
12	Fechter C Z Gerontol Geriatr 2020 [25]	2020	Germany	High	Quantitative	55+ workers	Health inequity	Employer flexibility	Longer working life; better health	Health-responsive FWAs

Note: ↑ means increase.

**Table 4:** Prevalence of Themes Among the 33 Included Studies.

Theme	Number of Studies Mentioning	Percentage (%)
Barriers	30/33	90.9%
Facilitators	30/33	90.9%
Outcomes	28/33	84.8%
Policy Implications	28/33	84.8%

**Table 5:** Barriers to Remote Work Among Older Adults.

Barrier Category	Number of Studies (*n* = 33)	Key Description
Digital exclusion/tech fatigue	14	Limited digital skills, lack of access to devices or reliable internet, and ICT-related strain hamper participation.
Ageism/employer bias	8	Stereotypes that older workers are less adaptable and lack technology skills, combined with a lack of age-friendly HR practices and support, contribute to this issue.
Health limitations/cognitive strain	8	Poor health, chronic conditions, or age-related cognitive decline reduce the capacity to work remotely.
Ergonomic/home-work challenges	2	Inadequate home workstations cause physical discomfort or musculoskeletal issues.
Organizational culture/role clarity	3	High job demands, unclear roles, limited social support, and unsupportive work cultures can undermine the success of remote work.
Regulatory/policy barriers	6	Legal or pension constraints hinder the adoption of phased retirement and formal telework, primarily due to the lack of national legislation or social insurance coverage for telework.

**Table 6:** Facilitators of Remote Work Among Older Adults.

Facilitator Category	Number of Studies (*n* = 33)	Key Description
Flexible scheduling/contract work	17	Part-time work, hybrid schedules, contract or self-employment options enable older adults to balance their health, caregiving, and work responsibilities.
Personalized arrangements (I-deals)	5	Tailored work arrangements that recognize individual needs and preferences (also known as idiosyncratic deals) increase motivation and retention.
Digital literacy/cognitive training	10	Structured digital skills training, cognitive upskilling, and mentoring programs help close digital gaps and reduce age-related barriers.
Sense of community/social support	18	Strong team cohesion, supportive leadership, clear communication, and peer support foster engagement and job satisfaction.
Good health/self-regulation	6	Autonomy, self-regulatory strengths, and good physical health enable productive remote work.
Policy and organizational support	10	Supportive policies (phased retirement, social insurance), ergonomic equipment, and inclusive HR practices facilitate sustained labor-force participation.

## Data Availability

No new data were created or analyzed in this study. Data sharing was not performed in this study.

## Supplementary Materials

The following supporting information can be downloaded at: https://www.mdpi.com/article/10.3390/ijerph22111719/s1. File S1: Full Search Strategy. File S2: Covidence Screening Documentation. File S3: Data Extraction Sheet. File S4: Completed PRISMA-ScR Checklist. All supplementary files and documentation related to this scoping review have been archived and made openly accessible via the Open Science Framework (OSF): https://osf.io/tdgsv (accessed on 23 September 2025). https://doi.org/10.17605/OSF.IO/B6H7C (accessed on 23 September 2025).

## References

[1] United Nations, Department of Economic and Social Affairs, Population Division World Population Ageing 2020 Highlights. https://www.un.org/development/desa/pd/sites/www.un.org.development.desa.pd/files/undesa_pd-2020_world_population_ageing_highlights.pdf.

[2] World Health Organization (2020). Decade of Healthy Ageing: Baseline Report.

[3] Eurofound, International Labour Office (2017). Working Anytime, Anywhere: The Effects on the World of Work.

[4] Eurofound (2021). Living, Working and COVID-19. https://www.eurofound.europa.eu/en/publications/2021/living-working-and-covid-19-update-april-2021-mental-health-and-trust-decline.

[5] Neves B.B., Amaro F. (2012). Too old for technology? How the elderly of Lisbon use and perceive ICT. J. Community Inform..

[6] Van der Lippe T., Lippényi Z. (2020). Co-workers working from home and individual and team performance. New Technol. Work Employ..

[7] Mann S., Holdsworth L. (2003). The psychological impact of teleworking: Stress, emotions and health. New Technol. Work Employ..

[8] Vyas L., Butakhieo N. (2021). The impact of working from home during COVID-19 on work and life domains: An exploratory study on Hong Kong. Policy Des. Pract..

[9] World Health Organization (1986). Ottawa Charter for Health Promotion.

[10] Keating N. (2022). A research framework for the United Nations Decade of Healthy Ageing (2021–2030). Eur. J. Ageing.

[11] Peters M.D.J., Godfrey C., McInerney P., Munn Z., Tricco A.C., Khalil H., Aromataris E., Munn Z. (2020). Chapter 11: Scoping Reviews (2020 version). JBI Manual for Evidence Synthesis.

[12] Tricco A.C., Lillie E., Zarin W., O’Brien K.K., Colquhoun H., Levac D., Moher D., Peters M.D.J., Horsley T., Weeks L. (2018). PRISMA Extension for Scoping Reviews (PRISMA-ScR): Checklist and Explanation. Ann. Intern. Med..

[13] Levac D., Colquhoun H., O’Brien K.K. (2010). Scoping studies: Advancing the methodology. Implement. Sci..

[14] Abraham K.G., Hershbein B., Houseman S.N. (2021). Contract work at older ages. J. Pension Econ. Financ..

[15] Andreassi S., Monaco S., Salvatore S., Sciabica G.M., De Felice G., Petrovska E., Mariani R. (2021). To Work or Not to Work, That Is the Question: The Psychological Impact of the First COVID-19 Lockdown. Healthcare.

[16] Arvola R., Tint P., Kristjuhan Ü., Siirak V. (2017). Impact of telework on the perceived work environment of older workers. Sci. Ann. Econ. Bus..

[17] Beekman E.M., Van Hooff M.M.L., Adiasto K., Claessens B.J.C., Van der Heijden B.I.J.M. (2025). Is This (Tele)working? A path model analysis of the relationship between telework, job demands and job resources, and sustainable employability. Work (Read. Mass.).

[18] Bal P.M., Jansen P.G.W., Bal P.M. (2014). Idiosyncratic deals for older workers. Aging Workers and the Employee-Employer Relationship.

[19] Buonomo I., Ferrara B., Pansini M., Benevene P. (2023). Job Satisfaction and Perceived Structural Support. Int. J. Environ. Res. Public Health.

[20] Czaja S.J., Sharit J. (2009). Aging and Work: Issues and Implications in a Changing Landscape.

[21] Damman M., Henkens K. (2020). Gender differences in workplace flexibility among older workers. J. Appl. Gerontol..

[22] Seifert A., Cotten S.R., Xie B. (2021). A double burden of exclusion? Digital and social exclusion of older adults in times of COVID-19. J. Gerontol. B Psychol. Sci. Soc. Sci..

[23] Dijkstra K. (2024). Future work skills for older workers. Gerontechnology.

[24] Dropkin J., Moline J., Kim H., Gold J.E. (2016). Blended Work as a Bridge Between Traditional Workplace Employment and Retirement: A Conceptual Review. Work Aging Retire..

[25] Fechter C. (2020). The role of health in flexible working arrangements in Germany. Z. Für Gerontol. Und Geriatr..

[26] Francis-Levin J., Webster N.J., Brauer S.G., Armstrong T.J., Antonucci T.C. (2024). Experiences and contexts of remote work among older, mid-life and young adults: The case for age-specific remote work interventions. Gerontechnology.

[27] Oakman J., Kinsman N., Stuckey R., Graham M., Weale V. (2020). A rapid review of mental and physical health effects of working at home. BMC Public Health.

[28] Chen M.K.L., Gardiner E. (2019). Supporting older workers to work: A systematic review. Pers. Rev..

[29] Hamouche S., Parent-Lamarche A. (2023). Teleworkers’ job performance: A study examining the role of age as an important diversity component of companies’ workforce. J. Organ. Eff..

[30] Hauret L., Martin L., Poussing N. (2024). Teleworkers’ digital up-skilling: Evidence from the spring 2020 lockdown. Inf. Soc..

[31] Koreshi S.Y., Alpass F. (2023). Understanding the use of Flexible Work Arrangements Among Older New Zealand Caregivers. J. Appl. Gerontol..

[32] Johnson R.W. (2011). Phased Retirement and Workplace Flexibility for Older Adults: Opportunities and Challenges. Ann. Am. Acad. Political Soc. Sci..

[33] Patrickson M. (2002). Teleworking: Potential employment opportunities for older workers?. Int. J. Manpow..

[34] Park S., Chaudhuri S., Johnson K.R. (2025). Engaging new hires in remote environments. Eur. J. Train. Dev..

[35] Hunter L.Y., Ginn M., Meares W.L., Hatcher W. (2024). Telework and Work Flexibility in the United States Federal Government Post-Pandemic. Public Adm. Q..

[36] Phuong T.T., Sukontamarn P. (2024). Effect of the COVID-19 pandemic on the employment and income of older workers in Vietnam. Asian Soc. Work. Policy Rev..

[37] Scheibe S., Retzlaff L., Hommelhoff S., Schmitt A. (2024). Age-related differences in the use of boundary management tactics when teleworking: Implications for productivity and work-life balance. J. Occup. Organ. Psychol..

[38] Oude Mulders J., Henkens K., van Dalen H.P. (2020). How Do Employers Respond to Aging Workforce?. Trends in Aging and Work.

[39] Kim J. (2024). Pandemic-Induced Telework Divide of Federal Workforces. Public Pers. Manag..

[40] Shifrin N.V., Michel J.S. (2021). Flexible work arrangements and employee health: A meta-analytic review. Work Stress.

[41] Zhao H., Xie H. (2025). Workplace Intelligent Technology Use and Health of Older Remote Workers. J. Occup. Environ. Med..

[42] Spoladore D., Trombetta A. (2023). Ambient Assisted Working Solutions for the Ageing Workforce: A Literature Review. Electronics.

[43] Foster-Thompson L., Mayhorn C.B., Hedge J.W., Borman W.C. (2012). Aging Workers and Technology. The Oxford Handbook of Work and Aging.

[44] Vanajan A., Bültmann U., Henkens K. (2019). Health-related Work Limitations Among Older Workers—The Role of Flexible Work Arrangements and Organizational Climate. Gerontologist.

[45] Wang C., Zhang Y., Feng J. (2024). How do older employees achieve successful ageing at work through generativity in the digital workplace? A self-affirmation perspective. J. Occup. Organ. Psychol..

[46] Wissemann M., Pit S., Serafin P., Gebhardt H. (2022). Strategic Guidance and Technological Solutions for Human Resources Management to Sustain an Aging Workforce: Review of International Standards, Research, and Use Cases. JMIR Hum. Factors.

[47] Koolhaas W., van der Klink J.J., de Boer M.R., Groothoff J.W., Brouwer S. (2014). Chronic health conditions and work ability in the ageing workforce: The impact of work conditions, psychosocial factors and perceived health. Int. Arch. Occup. Environ. Health.

[48] International Labour Organization (2020). Teleworking During the COVID-19 Pandemic and Beyond: A Practical Guide.

[49] Bureau of Labor Statistics (2024). The rise in remote work since the pandemic and its impact on productivity. Beyond Numbers.

[50] Komp-Leukkunen K. (2023). A Life-Course Perspective on Older Workers in Workplaces Undergoing Transformative Digitalization. Gerontologist.

[51] Piroșcă G.I., Șerban-Oprescu G.L., Badea L., Stanef-Puică M.-R., Valdebenito C.R. (2021). Digitalization and Labor Market—A Perspective within the Framework of Pandemic Crisis. J. Theor. Appl. Electron. Commer. Res..

[52] Money A., Hall A., Harris D., Eost-Telling C., McDermott J., Todd C. (2024). Barriers to and facilitators of older people’s engagement with web-based services: Qualitative study of adults aged > 75 years. JMIR Aging.

[53] OECD (2023). Disability, Work and Inclusion in Italy: Better Assessment for Better Support.

[54] Sharit J., Czaja S.J., Hernandez M.A., Nair S.N. (2009). The Employability of Older Workers as Teleworkers: An Appraisal of Issues and an Empirical Study. Hum. Factors Ergon. Manuf. Serv. Ind..

[55] (2022). Ageing Societies: General Requirements and Guidelines for an Age-Inclusive Workforce; 1st ed.

