# Supplementary material for: Remote Work, Well-Being, and Healthy Labor Force Participation Among Older Adults: A Scoping Review
Source: Int J Environ Res Public Health. 2025 Nov 13;22(11):1719. doi: 10.3390/ijerph22111719 (PMC12652596; doi:10.3390/ijerph22111719)
Supplement: Supplementary file 1 [file ijerph-22-01719-s001.zip › ijerph-3906388-supplementary/ijerph-3906388-supplementary-final/ijerph-3906388-supplementary-1/File S1 Full Search Strategy.pdf]

Remote Work and the Aging Workforce
Search strategies
Searches developed and conducted by Marie Ascher, Library Director, Phillip Capozzi, MD Library – May 2025

1. Database: Ovid MEDLINE(R) ALL <1946 to May 12, 2025>

1 aged.sh. 3654934
2 ((older adj3 worker*) or (older adj3 employee*) or (elderly adj3 worker*) or (elderly adj3 employee*) or (elderly adj3 personnel) or (older adj3 personnel)).ti,ab. 3182
3 ((employees adj2 aged adj "60") or (employees adj2 aged adj "65") or (employees adj2 aged adj "55") or (workers adj2 aged adj "60") or (workers adj2 aged adj "65") or (workers adj2 aged adj "55") or (personnel adj2 aged adj "60") or (personnel adj2 aged adj "65") or (personnel adj2 aged adj "55)).ti,ab. 4405
4 (later adj working adj life).ti,ab. 12
5 age factors.sh. 481134
6 teleworking.sh. 472
7 (flexib* adj2 workplace*).ti,ab. 128
8 telecommut*.ti,ab. 197
9 telework*.ti,ab. 685
10 ((hybrid adj2 work*) or (remote adj2 work*) or (virtual adj2 work*) or (online adj2 work*) or (work adj at adj home) or (work adj from adj home) or flexplace or (flexib* adj2 work* adj2 arrangement*) or (work* adj remote*)).ti,ab. 7310
11 1 or 2 or 3 or 43657217
12 6 or 7 or 8 or 9 or 10 8156
13 11 and 12 523
14 11 or 5 3953743
15 14 and 12 564

Date 5/12/25
Results: 564

2. Database: EMBASE

('telecommuting'/exp OR teleworking:ti,ab OR ((flexib* NEXT/2 workplace*):ti,ab) OR telecommut*:ti,ab OR ((hybrid NEAR/2 work*):ti,ab) OR ((remote NEAR/2 work*):ti,ab) OR ((virtual NEAR/2 work*):ti,ab) OR ((online NEAR/2 work*):ti,ab) OR ((work NEXT/1 from NEXT/1 home):ti,ab) OR ((work NEXT/1 at NEXT/1 home):ti,ab) OR flexplace:ti,ab OR ((work* NEAR/2 remote*):ti,ab))
AND

('aged'/exp OR ((older NEAR/3 worker*):ti,ab) OR ((older NEAR/3 employee*):ti,ab) OR ((older NEAR/3 personnel):ti,ab) OR ((elderly NEAR/3 worker*):ti,ab) OR ((elderly NEAR/3 employee*):ti,ab) OR ((elderly NEAR/3 personnel):ti,ab) OR ((employees NEXT/2 age* NEXT/1 '60'):ti,ab) OR ((employees NEXT/2 age* NEXT/1 '65'):ti,ab) OR ((employees NEXT/2 age* NEXT/1 '55'):ti,ab) OR ((workers NEXT/2 age* NEXT/1 '60'):ti,ab) OR ((workers NEXT/2 age* NEXT/1 '55'):ti,ab) OR ((workers NEXT/2 age* NEXT/1 '65'):ti,ab) OR ((personnel NEXT/2 age* NEXT/1 '60'):ti,ab) OR ((personnel NEXT/2 age* NEXT/1 '65'):ti,ab) OR ((personnel NEXT/2 age* NEXT/1 '55'):ti,ab) OR ((later NEXT/1 working NEXT/1 life):ti,ab))

Mapped termsn/a

Searched 5/13/25
RESULTS: 682

3. Database: SCOPUS
(TITLE-ABS-KEY ((telework*) OR (flexib* PRE/2 workplace*) OR (telecommut*) OR (hybrid W/2 work*) OR (remote W/2 work*) OR (virtual W/2 work*) OR (online W/2 work*) OR (work PRE/1 from PRE/1 home) OR (work PRE/1 at PRE/1 home) OR flexplace OR (work* W/2 remote*))) AND (TITLE-ABS-KEY ((aging W/2 worker*) OR (aging W/2 employee*) OR (aging W/2 personnel) OR (older W/2 worker*) OR (older W/2 employee*) OR (older W/2 personnel) OR (elderly W/2 worker*) OR (elderly W/2 employee*) OR (elderly W/2 personnel) OR (employees PRE/2 age* PRE/1 '60') OR (employees PRE/2 age* PRE/1 '65') OR (employees PRE/2 age* PRE/1 '55') OR (workers PRE/2 age* PRE/1 '60') OR (workers PRE/2 age* PRE/1 '55') OR (workers PRE/2 age* PRE/1 '65') OR (personnel PRE/2 age* PRE/1 '60') OR (personnel PRE/2 age* PRE/1 '65') OR (personnel PRE/2 age* PRE/1 '55') OR ("later working life")))

Results: 356
Date: 5/22/25

4. Database: CINAHL (EbscoHost)

(aged or "age factors" OR "older workers" OR "older employees" or "later working life" or "employees aged 50 or older" or "employees aged 55 or older" or "employees aged 60 or older" or "employees aged 65 or older" or "workers aged 50 or older" or "workers aged 55 or older" or "workers aged 60 or older" or "workers aged 65 or older") AND (telecommuting or teleworking or "working from home" or "remote work" or "flexible workplace" or telecommut* or telework* or "hybrid work" or "flexible work arrangements" or flexplace)

Results: 234
Date 5/22/25

5. Database: AGELINE (EbscoHost)
telecommuting or teleworking or "working from home" or "remote work" or "flexible workplace" or telecommut* or telework* or "hybrid work" or "flexible work arrangements" or flexplace
Limited to Academic journals and dissertations
Results: 56
Date 5/22/25

6. Database: PsycInfo (EbscoHost)
(aged or "age factors" OR "older workers" OR "older employees" or "later working life" or "employees aged 50 or older" or "employees aged 55 or older" or "employees aged 60 or older" or "employees aged 65 or older" or "workers aged 50 or older" or "workers aged 55 or older" or "workers aged 60 or older" or "workers aged 65 or older") AND (telecommuting or teleworking or "working from home" or "remote work" or "flexible workplace" or telecommut* or telework* or "hybrid work" or "flexible work arrangements" or flexplace)

Results: 166
Date: 5/22/15

TOTAL: 2058

7. Database: ECONLit
Econlit <1886 to May 29, 2025>

2 ((older adj3 worker*) or (older adj3 employee*) or (elderly adj3 worker*) or (elderly adj3 employee*) or (elderly adj3 personnel) or (older adj3 personnel)).ti,ab. 2085
3 (employees adj2 aged adj "60") or (employees adj2 aged adj "65") or (employees adj2 aged adj "55") or (workers adj2 aged adj "60") or (workers adj2 aged adj "65") or (workers adj2 aged adj "55") or (personnel adj2 aged adj "60") or (personnel adj2 aged adj "65") or (personnel adj2 aged adj "55”). 210
4 (later adj working adj life). 14382
5 "later working life".mp. 6
6 (telework* or (flexib* adj2 workplace) or telecommut*).mp. 659
7 ((hybrid adj2 work*) or (remote adj2 work*) or (virtual adj2 work*) or (online adj2 work*) or (work adj at adj home) or (work adj from adj home) or flexplace or (flexib* adj2

work* adj2 arrangement*) or (work* adj remote*)).mp. [mp=heading words, abstract, title, country as subject] 1274
8 (2 or 3 or 4 or 5) and (6 or 7) 40
Date: May 29, 2025
Results: 40

All uploaded to EndNote - Deduplicated total: 1793

60 more duplicates removed during import to COVIDENCE

Final deduplicated total = 1733

Remote Work and the Aging Workforce

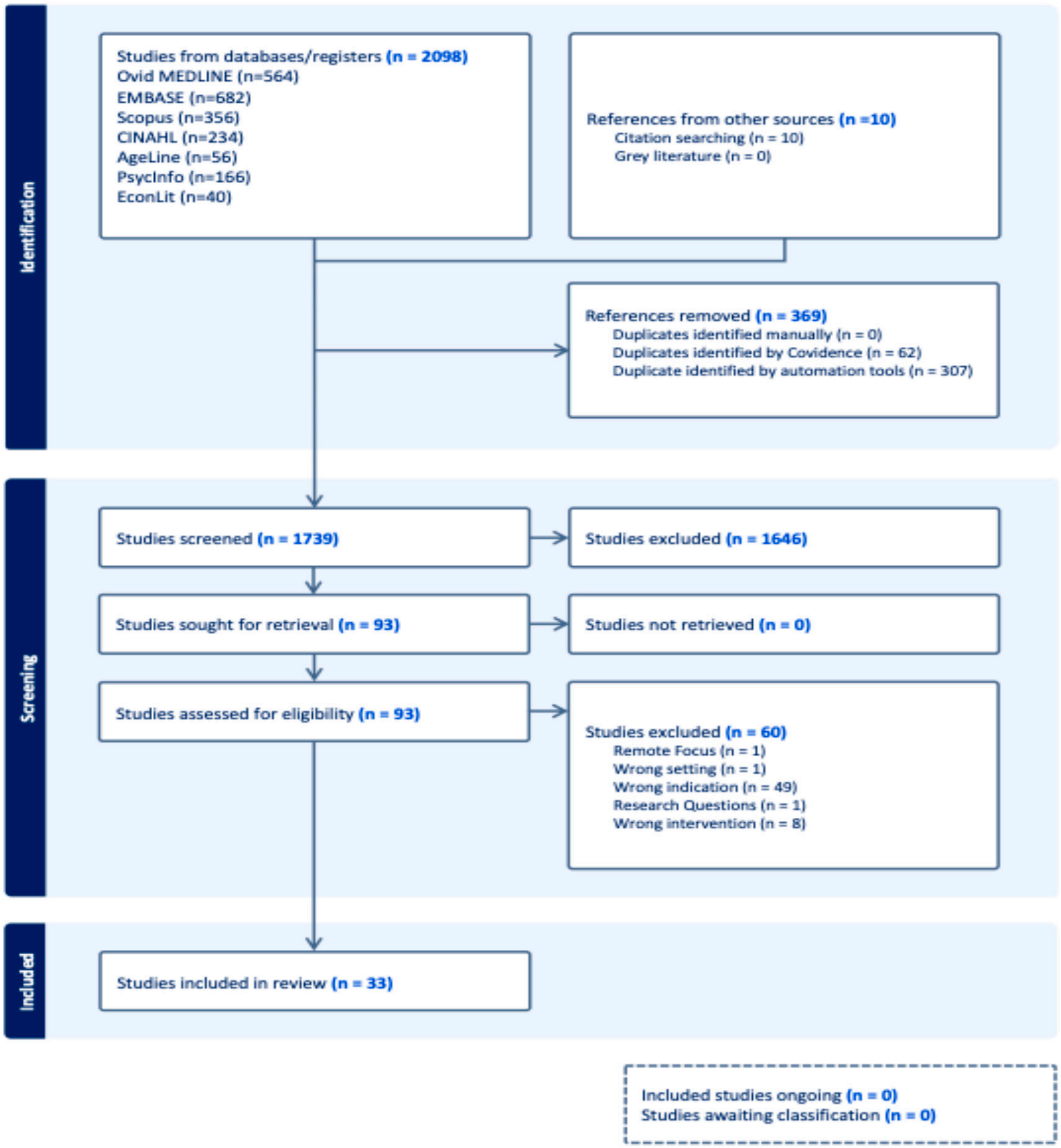

4th July 2025

covidence
