# Supplementary material for: Remote Work, Well-Being, and Healthy Labor Force Participation Among Older Adults: A Scoping Review
Source: Int J Environ Res Public Health. 2025 Nov 13;22(11):1719. doi: 10.3390/ijerph22111719 (PMC12652596; doi:10.3390/ijerph22111719)
Supplement: Supplementary file 1 [file ijerph-22-01719-s001.zip › ijerph-3906388-supplementary/ijerph-3906388-supplementary-final/ijerph-3906388-supplementary-1/File S3 Data Extraction Sheet.pdf]

Appendix B_1

	Study Title	Study Location	Study Design/Methods	Objective/Research Question	Population Characteristics	Remote Work Context	Barriers Identified	Facilitators Identified	Health Promotion and Labor Force Participation Outcomes	Policy implications
1	Contract Work at Older Ages<br><br>Abraham, K. G., Hershbein, B., & Houseman, S. N. (2021). *Contract work at older ages. Journal of Pension Economics and Finance*, 20(3), 426–447. https://doi.org/10.1017/S1474747220000098	United States (Gallup Survey nationally representative sample)	Quantitative analysis using Gallup Education Consumer Pulse Survey (n ≈ 61,000 adults, including ~40,000 aged 50+)	To understand how contract and self-employment work, especially as independent contractors, contributes to labor force participation among older adults	Adults aged 50–79; analysis stratified by age, education, gender, and occupation	Focus on independent contractors, some of whom work remotely (e.g., via platforms like Upwork, TaskRabbit); data includes platform-mediated work	Ageism in hiring.<br>Lack of employer support for phased retirement<br>Lower opportunities for independent contractor work among less-educated older adults	Flexibility of contract work<br>Option to work for former employer as a contractor (25% of older contractors)<br>Supplementing retirement income<br>High digital engagement among educated workers	Self-employment and contract work rise sharply with age: 67.5% of workers aged 75–79 were self-employed<br>Education strongly predicts continued work via independent<br>Contracting offers a bridge to retirement for many	Support flexible, contract-based models for older adults<br>Address educational gaps in access to contractor opportunities<br>Encourage phased retirement policies via former employers<br>Recognize self-employment as a critical post-retirement work form
2	To Work or Not to Work, That Is the Question<br>Andreassi, S.; Monaco, S.; Salvatore, S.; Sciabica, G.M.; De Felice, G.; Petrovska, E.; Mariani, R. To Work or Not to Work, That Is the Question: The Psychological Impact of the First COVID-19 Lockdown on the Elderly, Healthcare Workers, and Virtual Workers. *Healthcare*	Italy	Mixed-methods: quantitative (DERS, PTGI scales), qualitative (semi-structured interviews, linguistic analysis)	To investigate emotional regulation and post-traumatic growth in elderly, healthcare workers, and virtual workers during COVID-19 lockdown	257 participants: 62 elderly (mean age 71.7), 104 healthcare workers (mean age 35.3), 91 virtual workers (mean age 29.9)	Healthcare workers on-site, virtual workers remote, elderly retired or non-working	Emotional dysregulation, cognitive overload in virtual workers, isolation in elderly, burnout in healthcare workers	Spiritual growth in elderly, work engagement in healthcare workers, adaptive narrative processing	Virtual workers experienced greater emotional confusion; healthcare workers showed resilience; elderly showed greater affective processing	Need for targeted emotional support in remote work settings; enhance narrative and coping strategies; preserve work-private life boundaries

	2021, *9*, 1754. https://doi.org/10.3390/healthcare9121754									
3	Impact of telework on the perceived work environment Of older workers<br><br>Arvola R, Tint P, Kristjuhan Ü, Siirak V. Impact of telework on the perceived work environment of older workers. *Scientific Annals of Economics and Business*. 2017;64(2):199–214. doi:10.1515/saeb-2017-0013	Estonia	Quantitative survey-based study	To examine how telework affects older workers' perceptions of their work environment and overall satisfaction	Older workers, aged 50+, specific sample size not mentioned in abstract	Telework from home setting, voluntary or part of job flexibility	Limited ICT skills, ergonomic challenges at home, isolation	Flexible work hours, reduced commuting stress, personalized work environment	Telework can support longer workforce participation if physical and digital environments are supportive	Policy support needed for ICT training, ergonomic improvements, and social integration of remote older workers
4	Idiosyncratic deals for older workers: Increased heterogeneity among older workers enhance the need for I-deals<br><br>Bal PM, Jansen PGW. Idiosyncratic deals for older workers: increased heterogeneity among older workers enhance the need for I-deals. In: Bal PM, Kooij D, Rousseau DM, editors. *Aging Workers and the Employee-Employer Relationship*. Cham: Springer	Europe (general context, including UK and Netherlands)	Conceptual chapter with literature synthesis and theoretical model	To explore the need for individualized work arrangements (I-deals) due to increasing heterogeneity among older workers	Older workers (primarily Baby Boomers, aged 50+), discussed in general terms	Flexible and individualized work arrangements mentioned, including remote work as part of I-deals	Standardized HR practices, failure to recognize within-group heterogeneity among older workers	Individualized job arrangements (I-deals), flexible schedules, recognition of diverse older worker needs	I-deals can enhance older workers' motivation, productivity, and workforce retention when aligned with individual needs	Organizations should move toward individualized HR approaches to accommodate aging and heterogeneous workforce needs

	International Publishing; 2014. p. 129–144. doi:10.1007/978-3-319-08007-9_8									
5	IS THIS (TELE)WORKING? A path model analysis of the relationship between telework, job demands and job resources, and sustainable employability<br>Beekman, E.M., Van Hooff, M.M.L., Adiasto, K., Claessens, B.J.C., & Van der Heijden, B.I.J.M. (2025). *IS THIS (TELE)WORKING? A path model analysis of the relationship between telework, job demands and job resources, and sustainable employability*. WORK, 80(1), 295–313. https://doi.org/10.3233/WOR-240033	Netherlands (public/government sector)	Quantitative cross-sectional survey using path model analysis; sample size: 552 employees; conducted during COVID-19 lockdowns	To explore the relationship between teleworking, job demands/resources (work pressure, role clarity, social support, workplace communication), and sustainable employability (vitality, work ability, employability)	552 Dutch government employees; 46.3% male, 53.5% female, avg. age 49.7 (range 18–66); high education level (70% university-level)	Widespread mandatory telework during COVID-19; average telework % measured; data collected April–May 2021	Increased work pressure with more telework<br>Reduced role clarity<br>Minimal impact on social support and workplace communication	Role clarity and workplace communication linked positively to vitality and employability<br>Moderate work pressure may be motivational rather than detrimental	Telework associated with higher work pressure **and** lower role clarity<br>Work pressure unexpectedly linked to higher vitality<br>Role clarity **and** communication linked to higher employability and vitality<br>Minimal connection to sick leave/work ability (possibly due to COVID-era presenteeism)	Promote role clarity in telework settings (e.g., balanced scorecards, structured onboarding)<br>Monitor work pressure to prevent negative stress<br>Encourage communication structures to maintain employability<br>Recognize potential for presenteeism and monitor health appropriately
6	Job Satisfaction and Perceived Structural Support in Remote Working Conditions—The Role of a Sense of Community at Work	Italy (national sample)	Quantitative cross-sectional design; structural equation modeling (SEM); N = 635 remote workers	To examine whether perceived structural support and sense of community mediate the relationship between job demands and job satisfaction	635 participants; aged 21–70 (mean age = 46.7); 61% female; 33% had remote work experience before COVID-19 lockdown	Remote work during the COVID-19 pandemic; assessed prior experience and support structures; wide sectoral inclusion	High job demands under remote work<br>Lack of structural support had no significant buffering effect<br>Challenges in maintaining autonomy and dealing with ICT	Strong sense of community at work was a partial mediator, positively linked to job satisfaction<br>Social relationships and connection with coworkers	Job demands negatively affected job satisfaction<br>Sense of community mediated the relationship between job demands and job satisfaction<br>Structural support	Foster a sense of community even in remote environments through relational support strategies<br>Structural supports alone (e.g., equipment or training) may be insufficient without relational

	Buonomo, I., Ferrara, B., Pansini, M., & Benevene, P. (2023). *Job Satisfaction and Perceived Structural Support in Remote Working Conditions—The Role of a Sense of Community at Work. International Journal of Environmental Research and Public Health*, 20(13), 6205. https://doi.org/10.3390/ijerph20136205			among remote workers				mattered significantly	was not a significant mediator<br>Highlights importance of social connectedness for sustainable remote work	engagement<br>Design remote work policies that incorporate social infrastructure, not just technical
7	Aging and Work: Issues and Implications in a Changing Landscape<br><br>Czaja SJ, Sharit J, editors. *Aging and work: issues and implications in a changing landscape*. Baltimore (MD): Johns Hopkins University Press; 2009. 456 p. Reviewed by: Immerfall S. *Book Rev. J Aging Soc Policy*. 2012;24(4):413–414. doi:10.1080/10848770.2012.673351.	Primarily United States (though implications for Europe are discussed)	Edited volume containing 17 chapters, largely empirical and policy-based; data sourced from the Center for Research and Education on Aging and Technology Enhancement (CREATE), sponsored by the National Institutes of Health (NIH); mix of literature reviews, program evaluations, and policy analyses	To explore key challenges and opportunities in relation to work and the aging population, covering demographics, work design, cognitive demands, health, telework, entrepreneurship, and policy innovation	Primarily focuses on older workers (aged 50+) in the United States; includes discussions on age-related variation in performance, learning, health, and work attitudes; addresses needs of older adults in various sectors and organizational contexts	Addressed in the editors' chapter on **telework** highlights that how new work arrangements are implemented (not just their presence) determines success; telework may alleviate or worsen cognitive demands based on technology used	Structural inertia in workplace practices and social policy<br>Managerial biases about creativity and adaptability of older workers<br>Inadequate wellness and health programs for older adults<br>Higher "discount rates" for new learning in older adults (perceived lower ROI)<br>Lack of longitudinal data to understand attrition effects	Entrepreneurship as a late-career option<br>Shorter tenures offering flexibility attractive to older workers<br>Technological design and training tailored to older users can enhance cognitive performance<br>Holistic policy and organizational support (e.g., Finnish model) improves work ability	Older adults are capable of continued productivity, innovation, and contribution given the right supports. Current systems favor early retirement, but this model is becoming unsustainable<br>Flexibility, recognition, and lifelong learning incentives are essential to retaining older workers.	Urges comprehensive change at individual, organizational, and societal levels to extend working life. Recommends shifting away from outdated retirement paradigms, embracing hybrid work, redesigning training models, and addressing workplace bias. The book serves as a multidisciplinary call to action for policymakers, employers, and researchers.
8	Gender Differences in Perceived Workplace Flexibility Among	Netherlands	Quantitative survey-based analysis using 2015 data from the NIDI Pension Panel Study (N = 4,813),	To examine whether older male and female employees differ in perceived	Dutch older employees (60–65), working in government,	Location flexibility includes remote work possibilities	Women perceive significantly less workplace flexibility (both	Flexibility is associated with increased work satisfaction<br>- Human capital	Women had less perceived schedule and location flexibility	Flexibility is key to prolonging employment<br>Address gender inequities in

	Older Workers in the Netherlands: A Brief Report<br><br>Damman M, Henkens K. Gender differences in perceived workplace flexibility among older workers in the Netherlands: a brief report. *J Appl Gerontol*. 2020;39(8):915–921. doi:10.1177/0733464818800651		aged 60–65. Regression analysis used to identify predictors of workplace flexibility.	workplace flexibility, and how these differences may be explained.	education, care, and welfare sectors.	(e.g., working from home, on train, or at different sites). Subjective perceptions of such flexibility were central.	schedule and location)<br>Gender differences in job characteristics (e.g., education, sector, supervisory roles)<br>Lower prevalence of flexible roles in female-dominated sectors	(education, experience) and job roles (supervisory positions, occupational skill level) increase access to flexibility	The gender gap in location flexibility was fully explained by job-related and human capital differences<br>The gap in schedule flexibility persisted, indicating unmeasured factors like manager support or informal culture<br>Flexibility, particularly schedule flexibility, is positively correlated with late-career work satisfaction	flexibility access, especially in sectors employing more women<br>Customize retirement and job design policies to recognize and reduce these disparities
9	Future work skills for older workers<br>**Dijkstra, K. (2024).** ***Future work skills for older workers*. Gerontechnology, 23(2), 1–1.**<br>DOI: 10.4017/gt.2024.23.s.962.opp	Netherlands	Randomized controlled trial with pretest-posttest design. Participants (age 50+) completed six online cognitive training sessions targeting attention, memory, and flexibility. Comparison between training vs. general information control group.	To assess whether online cognitive training improves cognitive domains critical to sustainable employability among older workers, compared to passive information-only learning.	Adults aged 50+, recruited via social media; randomized into cognitive training vs. control group.	Not directly addressed; however, outcomes (attention, memory, flexibility) are highly relevant for digital and remote work environments. The training is **delivered online**, modeling digital adaptability.	Age-related cognitive decline (e.g., in working memory, flexibility, attention)<br>Challenges of modern workplace (open-plan offices, digital overload)<br>Potential under-preparation for cognitive demands of extended work life	Online training targeting key cognitive skills<br>Improvements in **verbal/numerical working memory**, **flexibility**, and **attention** through practice and feedback<br>Training available before significant cognitive decline begins	Significant improvements in trained group in: verbal working memory ($F(1,39)=4.18$, $p=.048$), flexibility ($F(1,39)=11.63$, $p=.002$), and attention ($F(1,39)=10.21$, $p=.003$)<br>Both groups improved in some tasks, but training group showed **greater gains**, suggesting that cognitive upskilling is a promising tool for sustaining	Proactive interventions (e.g., cognitive training) can **delay or reduce cognitive decline**<br>- Digital and cognitive adaptability training should be integrated into lifelong learning policies<br>Targeted programs can help older workers thrive longer in cognitively demanding environments

									employability past age 50	
10	Blended Work as a Bridge Between Traditional Workplace Employment and Retirement: A Conceptual Review<br><br>Dropkin J, Moline J, Kim H, Gold JE. Blended Work as a Bridge Between Traditional Workplace Employment and Retirement: A Conceptual Review. New Solut. 2021;30(4):270–282. doi:10.1093/workar/waw017	United States	Conceptual review integrating occupational health literature, policy analysis, and workforce trends	To define and explore the concept of 'blended work' as a transitional model between full employment and retirement for older workers	Older adults nearing retirement, aged approximately 50+ across occupational settings	Blended work includes flexible, remote, part-time, and non-traditional job formats adapted for older workers	Insufficient regulatory frameworks, job inflexibility, health constraints, lack of supportive organizational policies	Telework technology, supportive policy environments, ergonomic and cognitive accommodations, flexible hours	Blended work may enhance older adults' ability to remain in the labor force by aligning job demands with aging-related needs	Encourage development of blended work models, revise retirement and disability policies, and promote workplace flexibility and health accommodation
11	The role of health in flexible working arrangements in Germany: Avenues to a longer working life?<br><br>**Fechter C.** The role of health in flexible working arrangements in Germany: Avenues to a longer working life? *Z Gerontol Geriat.* 2020;53(4):334–339. doi: 10.1007/s00391-019-01551-1	Germany	Quantitative analysis using data from the German Ageing Survey (DEAS)	To examine the relationship between health and access to flexible work arrangements, and their impact on extending working life	German workers aged 55+	Focus on flexible working time and place, including telework and part-time options	Poor health, lower socioeconomic status, limited access to flexible work in some job sectors	Better health, employer flexibility, higher occupational status	Healthier older workers benefit more from flexible arrangements; health influences both access to and benefits from such work models	Health promotion and targeted flexibility policies could help extend work life, especially for lower-status workers
12	Experiences and contexts of remote work among older, mid-life and young adults: The case for agespecific remote work interventions	U.S. sample from the Survey of Consumer Attitudes (SCA) confirms the setting	The article uses a quantitative survey analysis (cross-sectional), appropriately listed	Precisely reflects the paper's aim: to identify age-specific differences in remote work experience	Covers ages 18–44, 45–64, and 65+, with proper focus on older adults (aligned	Conducted during COVID-19 with participants who worked	Younger remote workers more likely to report stress and social isolation	Older and mid-life adults experienced less stress, possibly due to life stage and work experience	Older adults reported significantly lower stress, supporting age-specific intervention	Develop age-tailored interventions to support well-being in remote work environments across the lifespan

	Francis-Levin J, Webster NJ, Brauer SG, Armstrong TJ, Antonucci TC. Experiences and contexts of remote work among older, mid-life and young adults: the case for age-specific remote work interventions. *Opp: Work, Leisure, & Social Participation*. 2022 Dec–2023 Jan				with your 45+ inclusion)	remotely part- or full-time			development for remote work	
13	Supporting older workers to work: a systematic review Chen MKL, Gardiner E. Supporting older workers to work: a systematic review. *Personnel Review*. 2019;48(5):1318–35. doi:10.1108/PR-11-2018-0455.	International (majority Western countries)	Systematic quantitative literature review of 27 studies (1995–2016)	What work-related factors influence older workers' decisions to continue workforce participation?	Older workers aged 50+ across healthcare and social assistance sectors	Not specifically focused on remote work; study examined general workplace factors	Limited flexible work arrangements, salary not a strong motivator	Job autonomy, development opportunities, recognition, mentoring, supportive climate, social support	Participation influenced by psychosocial work factors; not by salary or flexibility alone	Organizations and governments should enhance meaningful work, autonomy, and support structures to retain older workers
14	Hamouche, S. and Parent-Lamarche, A. (2023), "Teleworkers' job performance: a study examining the role of age as an important diversity component of companies' workforce", Journal of Organizational Effectiveness: People and Performance, Vol. 10 No. 2, pp. 293-311. https://doi.org/10.1108/JOEPP-03-2022-0057	Canada	Quantitative study using multivariate and moderation regression analyses	To examine the direct effects of teleworking and age on job performance and to analyze the moderating effect of age on the relationship between teleworking and in-role job performance	272 employees across 18 companies; includes both older and younger workers	Telework	Older workers show reduced performance during teleworking due to age-related challenges with technology	Decision, authority, and recognition	Teleworking: Older age is associated with lower job performance; younger age is linked to higher age. Performance. On-site work: Older age linked to higher job performance, younger age to lower performance	Highlights the need for age-inclusive work arrangements; managers should adopt flexible, supportive telework policies that address age-specific needs

15	Hauret, L., Martin, L., & Poussing, N. (2024). Teleworkers' digital up-skilling: Evidence from the spring 2020 lockdown. *The Information Society*, *40*(3), 215–231. https://doi.org/10.1080/01972243.2024.2333025	Luxembourg	Cross-sectional and regression analysis	To assess whether the COVID-19 lockdown supported teleworkers by improving their digital skills and whether traditionally disadvantaged groups including older workers experienced a digital skills catch-up	438 employees	Telework	Lower digital skills among less educated;	Training, peer influence, necessity of remote work, learning-by-doing	Digital skills improved for women and older workers. Gender and age gaps narrowed, but the educational gap widened	Employers should offer targeted digital training, especially for the less educated, to prevent long-term inequality in digital competencies in hybrid and remote work settings
16	Koreshi, S. Y., & Alpass, F. (2023). Understanding the use of Flexible Work Arrangements Among Older New Zealand Caregivers. *Journal of applied gerontology: the official journal of the Southern Gerontological Society*, *42*(5), 1045–1055. https://doi.org/10.1177/07334648231152153	New Zealand	Quantitative study, Hierarchical regression analysis of 2018 Health, Work and Retirement survey data	To examine whether older caregivers use flexible work arrangements (FWAs) more than non-caregivers and what explains any differences	1,907 paid workers aged 55–70 years, including 296 caregivers and 1,611 non-caregivers	Flexibility in hours, schedules, and time off	Caregiving demands not matched by workplace support	Availability of FWAs	Caregivers use more FWAs than non-caregivers across multiple types of flexibility	Highlights the need for organizational and government policies to support older working caregivers, especially to improve women's workforce participation and promote prolonged employment
17	Johnson, R. W. (2011). Phased Retirement and Workplace Flexibility for Older Adults: Opportunities and Challenges. *The ANNALS of the American Academy of Political and Social Science*, *638*(1), 68-85. https://doi.org/10.1177/0002716211413542 (Original work published 2011)	United States	Conceptual and Policy analysis	To explore the opportunities, challenges, and policy barriers surrounding phased retirement and workplace flexibility for older adults	Older workers aged 50+; focus on white- and blue-collar workers across industries;	FWAs-Part-time, flexible schedules and telework	In-service pension distribution limits; benefit loss for part-time workers; tax code nondiscrimination rules; litigation risk; administrative complexity.	Older worker interest; employer need to retain skills; informal phased retirement practices	Formal phased retirement programs are difficult to implement While many employers are willing to offer informal phased retirement, legal, regulatory, and benefit-related hurdles prevent widespread adoption of formal programs. Older workers	Policy reforms to relax pension access rules, clarify age discrimination risks, and adjust nondiscrimination standards to encourage broader access to phased retirement—especially for lower-paid workers. Highlights the urgency of adapting workplace policies to an aging labor force

									prefer flexible schedules to manage caregiving, health, or stamina issues. Formalization is rare, and benefits are often withheld from part-timers	
18	Patrickson, M. (2002), "Teleworking: potential employment opportunities for older workers?", *International Journal of Manpower*, Vol. 23 No. 8, pp. 704-715. https://doi.org/10.1108/01437720210453902	Australia	Conceptual/Theoretical analysis	To assess whether teleworking opens work opportunities for older workers and historically marginalized in employment	Older workers in Australia	Telework	Employer attitudes, lack of hiring practices;	Potential suitability of older workers for teleworking<br><br>Growing tech capacity<br>Policy support for flexible work	Need for policy and employer practice reforms to reduce bias and increase access to telework for older, marginalized populations	Need for policy and employer practice reforms to reduce bias and increase access to telework for older, marginalized populations
19	Park, S., Chaudhuri, S. and Johnson, K.R. (2025), "Engaging new hires in remote work environments: exploring the challenges and opportunities in different employee age groups", *European Journal of Training and Development*, Vol. ahead-of-print No. ahead-of-print. https://doi.org/10.1108/EJTD-10-2024-0146	Not location specific conceptual paper	Conceptual approach; framework development	To explore the importance of new hire engagement in remote work for younger and older employees and propose a framework for support	45 years and above	Work from home and telework	Tech learning gaps for older workers, social isolation, unclear role expectations, limited digital support.	Tech-savviness for younger workers, autonomy for older workers digital onboarding tools, mentoring, organizational support	Older generations value independence but require support for digital adaptation. Engagement depends on strong onboarding, communication, and tailored support	Organizations should adopt a six-component framework (socialization, adaptation, communication, relationships, job/work design, and support) to foster engagement. Age-inclusive onboarding and mentoring policies are essential for maximizing productivity and retention in remote work environments
20	Hunter, L. Y., Ginn, M., Meares, W. L., & Hatcher, W. (2024). Telework and Work Flexibility in the United States	United States	Quantitative logistic regression	To examine whether telework and workplace flexibility influence federal employees'	U.S federal workforce Primarily older workforce; <7% under	Telework spiked to 45% in 2020	Political opposition, unequal access, risk of isolation.	Flexible supervisors, telework eligibility, agency support	Access to telework and flexible supervisors significantly reduce turnover	Continued investment in teleworking and workplace flexibility is critical for retaining older

	Federal Government Post-Pandemic. *Public Administration Quarterly*, *48*(3), 149-161. https://doi.org/10.1177/07349149241231096 (Original work published 2024)			intentions to stay post-COVID	30; nearly 1/3 approaching retirement;			during COVID-19	intention; flexible work helps retain talent and combat burnout	workers, recruiting younger talent, and supporting a resilient federal workforce
21	Effect of the COVID-19 pandemic on the employment and income of older workers in VietnamTrieu Thi Phuong, Pataporn Sukontamarn 2024<br><br>https://doi.org/10.1111/aswp.12307	Vietnam	Secondary analysis of nationally representative 2021 Labor Force Survey; multinomial logistic regressions	Identify demographic & employment factors linked to COVID-related changes in work status (job loss, reduced hours, remote shift) and income among workers aged ≥50.	Identify demographic & employment factors linked to COVID-related changes in work status (job loss, reduced hours, remote shift) and income among workers aged ≥50.	222,413 adults ≥50 in LFS	Informal/self-employment; low skills/education; limited IT access; sector shocks (services, industry); urban lockdowns; lack of social insurance.	Formal/state/private/FDI employment; social insurance; higher education; prior IT use enabling remote/online work	74.9% reported no employment change; 22.9% temp absence/reduced hours; 1.6% job loss; 31.6% income reduction. Formal jobs & social insurance lowered risks of job & income loss; education & IT use increased odds of remote work and buffered employment shocks; higher education reduced income loss. Older age groups generally had lower relative risk of job loss than 50–54 group.	Formalize employment relationships; expand social insurance coverage; invest in digital/IT upskilling for older workers; promote pathways to remote work; sector/region-targeted supports during shocks; strengthen protections for vulnerable informal older workers to bolster late-life income security.
22	Oude Mulders, J., Henkens, K., van Dalen, H.P. (2020). How Do Employers Respond to an Aging Workforce? Evidence from Surveys Among Employers, 2009–2017. In: Czaja, S., Sharit, J., James, J. (eds) Current and Emerging Trends in	Netherlands	Longitudinal panel survey	To examine changes in employer practices, perceptions, and policies toward older workers over time.	Employers across sectors employing older workers	Flexible working arrangements	Productivity concerns, preference for hiring women, technological substitution	Increased training, flexible work hours, ergonomic support	Shift from exit-based to accommodative strategies; rise in training (8% → 40%), flexible work options; norms shifted to later retirement; low older worker hiring (10%) continues	Support for part-time retirement, phased retirement, demotion; policies for physical-demand job relief; call for public-private age-friendly labor policies

	Aging and Work. Springer, Cham. https://doi.org/10.1007/978-3-030-24135-3_14									
23	Kim J. (2023). Pandemic-Induced Telework Divide of Federal Workforces. *Public Personnel Management*, 00910260231175129. https://doi.org/10.1177/00910260231175129	United States	Quantitative analysis regression analysis	Examine how pandemic-induced telework experiences differed across federal employees and impacted engagement	U.S. federal employees; diverse in age, gender, disability status, and ethnicity	Telework	Disparities by age, disability, ethnicity, and gender.	Leader and organizational support (effective for gender only	71% increase in telework; disparities persisted by demographic group; leader /organizational support reduced gender disparity but not others	Develop inclusive strategies and supportive environments to improve telework access for older, disabled, and minority employees
24	Age and health jointly moderate the influence of flexible work arrangements on work engagement: Evidence from two empirical studies Journal of Occupational Health Psychology 2017;22(1):40-58 2017	United States	Two empirical studies using survey data	This study examined how age and health jointly moderate the relationship between flexible work arrangements (FWAs) and work engagement.	Study 1: older employees over 40 years old. Study 2: older employees over 50 years old	flexible work arrangement includes telecommuting as a component of FWAs	Older employees in poorer health benefit less from FWAs, suggesting that age and declining health may limit the effectiveness of such policies.	Better functional health facilitate the positive effects of FWAs on work engagement. Access to and use of flexible work policies are also key enablers.	The study found that subjective and chronological age, along with physical and mental health, influence how employees engage with work under flexible arrangements.	Organizations should adopt age- and health-responsive flexibility policies. A one-size-fits-all approach to flexible work is suboptimal; tailoring FWAs to worker demographics and health status can enhance engagement and retention.
25	Age- related differences in the use of boundary management tactics when teleworking: Implications for productivity and work- life balance DOI: 10.1111/joop.12512	United Kingdom, Canada, South Africa	Quantitative longitudinal study	The study is looking at if Age is positively associated with the use of boundary management tactics to separate work and nonwork life domains when teleworking from home.	ages ranged from 20 to 69 years	telework	Younger employees may lack experience or strategies for segmenting work and personal life during telework. High work and home demands, and weak boundary preferences, can limit the successful use of these tactics.	Older age itself was a facilitator due to associated self-regulatory strengths, greater experience, and prioritization of well-being all of which enhanced boundary management capabilities.	Older teleworkers were more likely than younger ones to use segmentation-oriented boundary management tactics (e.g., physical separation, digital boundaries, structured communication, and scheduling). These tactics were associated with fewer	Cross-generational knowledge sharing should also be promoted to leverage older workers' experience in boundary setting.

									unfinished tasks (higher productivity) and better work–life balance. Age indirectly predicted better telework outcomes via increased use of such tactics.	
26	Retirement decisions in times of COVID-19: the role of telework, ICT-related strain and social support on older workers' intentions to continue working Personnel Review 2024;53(8):1950-1973<br>2024<br>DOI<br>10.1108/pr-04-2023-0286	Western-Europe	A two-wave longitudinal study	this study investigated the change in older workers' intention to continue working during COVID-19 and the role of ICT-related strain and social support during teleworking	50 years or older	Telework and remote work.	study highlights that ICT-related strain serves as a potential barrier to older workers' continued workforce participation. While the research provides valuable insights into how factors like social support may stimulate or encourage ongoing work engagement, the stress and challenges associated with technology use—particularly during telework—can negatively impact older workers' intention to remain employed, especially those nearing retirement.	Perceived social support at work (buffering role, although not significant in moderated mediation	Older workers nearing retirement experienced a decline in their intentions to keep working during the pandemic. In contrast, those further from retirement showed an increase in their intentions to continue working. a higher intensity of telework was negatively associated with intentions to continue working.<br>This negative relationship was mediated by ICT-related strain—meaning that the stress or difficulty related to information and communication technology (ICT) use played a key role in this link.	organizations can establish a telework-buddy system, connecting experienced teleworkers with those who have less experience, to facilitate effective remote work. Leaders in times of COVID-19 can provide sufficient resources to buffer job demands and overcome negative feelings, such as loneliness and isolation, that can push older workers toward retirement
27	The employability of older workers as	USA	Quantitative survey-based study.	To examine the employability of	50 to 65, 66 to 80	Home-based telework.	Barriers included the	Older workers were rated	Older workers are seen as strong in	Policies at both state and federal levels

	teleworkers: An appraisal of issues and an empirical study Human Factors and Ergonomics In Manufacturing 2009;19(5):457-477 2009		Internet questionnaire administered to 314 managers across diverse U.S. industries.	older workers as home-based teleworkers.<br><br>To assess managerial attitudes about older workers and telework-related attributes.<br><br>To identify facilitators and barriers that influence the adoption of telework for older employees.		Tasks suitable for telework: Data processing, customer service, accounting, etc.	ability of older workers to adapt to the technological demands that are typically associated with telework jobs and managerial attitudes about older workers and about telework	significantly higher than younger workers on Trustworthiness and realibility. Many older workers prefer part-time or flexible work, which telework naturally supports. This aligns with findings that older adults are increasingly interested in nontraditional employment models. Entities like the U.S. Department of Labor and European Commission have advocated flexible and phased retirement, with telework identified as a promising strategy to retain older talent.	key telework traits like trustworthiness, reliability, independence, and time management, especially by experienced managers. However, they are perceived as weaker in adaptability, teamwork, and technology skills, resulting in a mixed but nuanced outlook for older workers in remote roles.	are needed to promote telework for older workers, particularly in the private sector, and to ensure they are informed about such opportunities. Existing labor laws, focused on centralized workplaces, must be updated to address home-based work issues, such as employer responsibility for safety, liability, and privacy in performance monitoring. States might also offer tax credits to companies for providing telework equipment, as seen in Oregon (Oregon Department of Energy, 1995). legal uncertainties may hinder wider adoption of telecommuting.
28	Spoladore, D.; Trombetta, A. Ambient Assisted Working Solutions for the Ageing Workforce: A Literature Review. Electronics 2023, 12, 101. https://doi.org/10.3390/ electronics12010101	Global Perspective (USA, Europe, Thailand, Indonesia)	Systematic literature review using PRISMA methodology. Searched Web of Science, Scopus, and PubMed for English-language journal articles (2010–2022). From 1598 initial records, 55 papers were screened; 19 met revised	RQ1: Are there existing Ambient Intelligence (AmI) solutions specifically designed for the ageing workforce?<br>RQ2: What technologies are being used in these solutions?	workers aged 55 or older who may have physical and cognitive limitations, chronic conditions, loss of muscular strength,	Study doesn't specifically address remote work contexts. Most studies focused on in-person work environments such as offices, hospitals, mines, industrial setting,	The acceptance of new technologies in workplaces is pivotal, but to overcome barriers, especially among older workers, more effort should be put into explaining	AAW is aimed at supporting older workers in a variety of workplaces, helping them in different activities through the deployment of technologies that can range	No AmI systems were designed specifically for the ageing workforce.Many AmI solutions can be adapted to older workers, addressing safety, task support, and comfort.	There is an urgent need for dedicated AAW (Ambient Assisted Working) research. Recommend workplace policies and digital transformation strategies that are inclusive of older workers. Call for standardization of

			inclusion criteria (i.e., potentially applicable to older workers)	Modified RQ1a: Identify existing AmI solutions not originally designed for older workers but that could potentially support them.	and decline in vision and hearing.	Orchestra, and driver	and nurturing transformation acceptance (i.e., companies' processes, and workers' jobs may undergo significant changes)	from wearable and environmental sensors to cyber-physical systems, as well as involving artificial intelligence (AI) to Analyze data.		AAW solutions, Alignment with Industry 4.0 tools (though not consistently explored).
29	Aging Workers and Technology by Foster-Thompson & Mayhorn 2012; DOI: 10.1093/oxfordhb/9780195385052.013.0113	USA	Review Chapter	To highlight the dual role of emerging technologies in shaping the work experience of older adults. It emphasizes how technology can support older workers by compensating for age-related limitations and enhancing opportunities, while also acknowledging the potential challenges it can create, particularly around usability and acceptance.	Older workers (typically aged 55+)	Telecommute, Remote work.	Challenges faced by older workers often arise from poor technology usability and resistance to adopting new tools, due to attitudinal resistance to technology adoption.	Technology can offset physical and cognitive decline. Telecommuting and CAM systems Promotes safe, independent work.	The study advocates for thoughtful design and training to ensure technology facilitates, rather than hinders, older workers' success and reintegration into the workforce. Attention to these matters will advance the success of older adults who remain in the workforce and facilitate the reintegration of those who seek re-employment after retirement. Workforce trends show more adults aged 60+ are working, with 39% of those 50+ planning to work in retirement. Remote work and flexibility boost retention and re-entry.	No direct policy implication stated

30	Health-related work limitations among older workers—The role of flexible work arrangements and organizational climate<br>The Gerontologist 2020;60(3):450-459 2020<br>doi:10.1093/geront/gnz073	Netherlands	Cross-sectional analysis. This study used data from the #rst wave of the NIDI Pension Panel Survey conducted in 2015. sample size include: 5,419 older workers aged 60–65 years across 624 organizations. Multilevel ordered logistic regression models.<br><br>Variables: perceived access to flexible work arrangements (FWAs), organizational climate (healthy ageing and psychological safety), health-related work limitations.<br><br>Controlled for demographics, comorbidities, job-related factors.	To assess whether and how flexible work arrangements and organizational climates may help to reduce the work limitations experienced by older workers. Grounded on the Job Demand–Resource model, we hypothesize that access to flexible work arrangements (working-time flexibility, workplace flexibility, phased retirement) and supportive organizational climates (healthy ageing climate, psychological safety climate) are vital job resources that are associated with fewer health-related work limitations among older workers experiencing CHCs.	Aged 60–65 years	Flexible Work Arrangements (FWAs) assessed. (working-time flexibility, workplace flexibility, phased retirement) and supportive organizational climates (healthy ageing climate)	Barriers to managing chronic health conditions (CHCs) in the workplace include rigid work hours and physically demanding blue-collar roles. Older workers often face additional challenges due to comorbid CHCs and psychological disorders.	Flexible working hours are especially beneficial for those with arthritis and sleep disorders. A psychologically safe organizational climate is linked to fewer work limitations across all CHCs, while a healthy ageing climate particularly supports workers with sleep disorders.	The study shows that offering flexible working hours and ensuring a psychologically safe climate, where older workers with health issues are inclined to share their work needs and preferences, are likely to contribute to healthy ageing in the workplace.	Policymakers can Encourage age-inclusive HR practices and flexible work structures. Promote organizational interventions (e.g., climate training) to support healthy ageing at work. Facilitating longer working lives is a key policy challenge within organizations, in particular if<br><br>older workers are constraint by CHCs.
31	Wang, C., Zhang, Y., & Feng, J. (2024). How do older employees achieve successful ageing at work through generativity in the digital workplace? A self-affirmation perspective. Journal	China	mixed-method, multi-wave quantitative design	This study investigates how older employees achieve successful ageing at work through intergenerational knowledge sharing (IGKS) via enterprise	Study 1: older employees over 40 years old)<br><br>Study 2: older employees	telework/remote work and older adults in the context of digital workplaces	Barriers include age-related threats to self-integrity and a digital divide	facilitators include organizational support, positive self-perception, and younger colleagues' absorptive capacity.	findings reveal that IGKS in ESM improves job performance (task proactivity, in-role, and extra-role performance) by enhancing personal needs–job supplies fit	rganizations should support older workers in using digital platforms for generative behaviors and foster environments where younger workers are receptive learners.

	of Occupational and Organizational Psychology, 97(4), 1475–1501. https://doi.org/10.1111/joop.12525			social media (ESM), using a self-affirmation theory lens.	over 50 years old				and organization-based self-esteem. The study also shows that the absorptive capacity of younger colleagues facilitates this process, while high disseminative capacity can hinder it	
32	Strategic Guidance and Technological Solutions for Human Resources Management to Sustain an Aging Workforce: Review of International Standards, Research, and Use Cases. JMIR Human Factors, 9(3):e27250. https://doi.org/10.2196/27250	Global (International perspective with examples from Europe, Australia, USA, Japan, Korea, and Pakistan, also includes Asian and Latin American countries	literature review, includes international standards analysis, technology role, and applied use cases to create and maintain a sustainable aging workforce	(1) explore how technological solutions in organizations can help create and maintain a healthy, productive, and capable aging workforce; and (2) provide recommendations and strategic guidance that benefit both the aging worker and the organization.	55 years and older	remote work	Barriers to blockchain adoption include fear of layoffs, worker resistance, and lack of blockchain competence. Worldwide adoption, support, and funding. Also highlights digital illiteracy, and bias in AI. age, gender, and lower health status are barriers to sustainable employability	Digital literacy training and remote work support for an age-inclusive workforce. Use of international standards (e.g., ISO 25550) and ethical AI frameworks to design Age-inclusive technology development.	Age bias in artificial intelligence development in the workplace can be avoided through inclusive practices by Involving older people in the planning, development, and implementation of ICT applications. No blockchain application was found yet to improve the aging workforce. Recent tools such as the Ageing@Work toolkit consisting of virtual user models and virtual workplace models allow for the adaptation of the work processes and the ergonomics of workplaces to the	Organizations and governments should adopt international standards and ethical frameworks to implement age-inclusive technologies and sustain the aging workforce. Emphasis on co-design, upskilling, and avoiding AI bias is critical.

									evolving needs of aging workers.	
33	Workplace Intelligent Technology Use and Health of Older Remote Workers Hairong Zhao 1, Huier Xie doi: 10.1097/JOM.0000000000003402	Europe	Quantitative analysis study using secondary survey data. Based on real-world data from the Survey of Health, Ageing and Retirement in Europe- SHARE survey (Wave 8). This study analyzes the mental health of older employees in the context of automation application, clarifying the psychological impact of auto-mation technology on older employees	Investigate the effect of automation job (eg, artificial intelligence [AI]) has facilitated the organizational efficiency as well as imposed technical demands on older employees.	age 50 and older, which covers 28 European countries as well as Israel	Telework, Older remote workers	Older adults experience technophobia and cognitive strain from complex tech demands.	Remote work helps increase job flexibility and autonomy, through which their psychological burden and tension can be reduced. Overall improved work-life balance.	The findings suggest that remote work and digital technologies offer valuable opportunities to extend older employees' careers. Older workers show adaptability and maintain strong performance with proper training, while remote work provides autonomy and flexibility, making it an attractive option.	Enhancing mental health support for older employees is vital. Organizations should offer annual screenings and counseling services, while governments must strengthen pension and economic security and expand access to health services.
34	Siegenthaler, J. K., & Brenner, A. M. (2000). Flexible work schedules, older workers, and retirement. Journal of aging & social policy, 12(1), 19–34. https://doi.org/10.1300/j031v12n01_03	United States	Literature review	Examine how flexible work schedules influence older workers' decisions to retire or remain in the workforce	Older workers nearing or past traditional retirement age	Reduced hours, part-time, and phased retirement options	Structural disincentives in retirement law (e.g., pension rules), lack of part-time benefits, and risk of age discrimination	social science advocacy for phased retirement	Flexible schedules can delay retirement for some older workers, but are underutilized due to legal, financial, and administrative barriers. Formal programs are rare, and informal arrangements are inconsistent.	Reforming retirement policies and benefit structures could encourage phased retirement, support older worker retention, and reduce age-based disparities in flexible work access.
